# Different diversity-dependent declines in speciation rate unbalances species richness in terrestrial slugs

**DOI:** 10.1038/s41598-017-16417-y

**Published:** 2017-11-23

**Authors:** Regina L. Cunha, Cláudia Patrão, Rita Castilho

**Affiliations:** CCMAR (Centre of Marine Sciences) - Campus de Gambelas, Universidade do Algarve, 8005-139 Faro, Portugal

## Abstract

Two genera of terrestrial slugs (*Arion* and *Geomalacus*) display a striking disproportion in species richness in the Iberian Peninsula. While there are 17 Iberian endemic species in *Arion*, morphological criteria only recognize four species within *Geomalacus*. Sequence data were used to test whether these differences could result from: (1) cryptic diversity within *Geomalacus*; (2) an earlier origin for *Arion* (older clades are expected to accumulate more species); (3) distinct patterns of diversification rates (higher initial speciation rates in *Arion*), and (4) some combination of the above factors (e.g., an older clade with higher speciation rates). Species delimitation tests based on mitochondrial and nuclear data revealed eight cryptic lineages within *Geomalacus* that lessened the asymmetry; nevertheless, the disparity required further investigation. No meaningful differences in crown group ages of each recovered clade were found. Regardless the different premises of the two equally plausible diversification models (similar initial speciation rates *vs*. higher initial speciation rates in *Geomalacus*), both coincide on diversity-dependent diversification for the two groups but weaker rate declines in *Arion* best explains the observed asymmetry in species richness. Also, the broader environmental tolerance combined with a faster dispersal and wider distribution may have represented an evolutionary advantage for *Arion*.

## Introduction

Patterns of species richness are often unbalanced throughout the tree of life^1^. These disproportions may reflect differences in clade ages given the expectation that the number of species will increase through time, and therefore, older clades are expected to display more diversity, as long as extinction rates remain constant. Another possibility is the existence of shifts across phylogenies with clades evolving at different rates^2^. Differences in species richness across clades may, however, not reflect a real biodiversity bias. The existence of hidden diversity within an apparently species-poor clade may create an artificial disequilibrium. Cryptic species, defined as two morphologically indistinguishable taxa that are classified as a single nominal species^3^, represent a significant component of total biodiversity that is often underestimated when taxonomy relies exclusively on morphological characters^4^.

**Figure 1:**
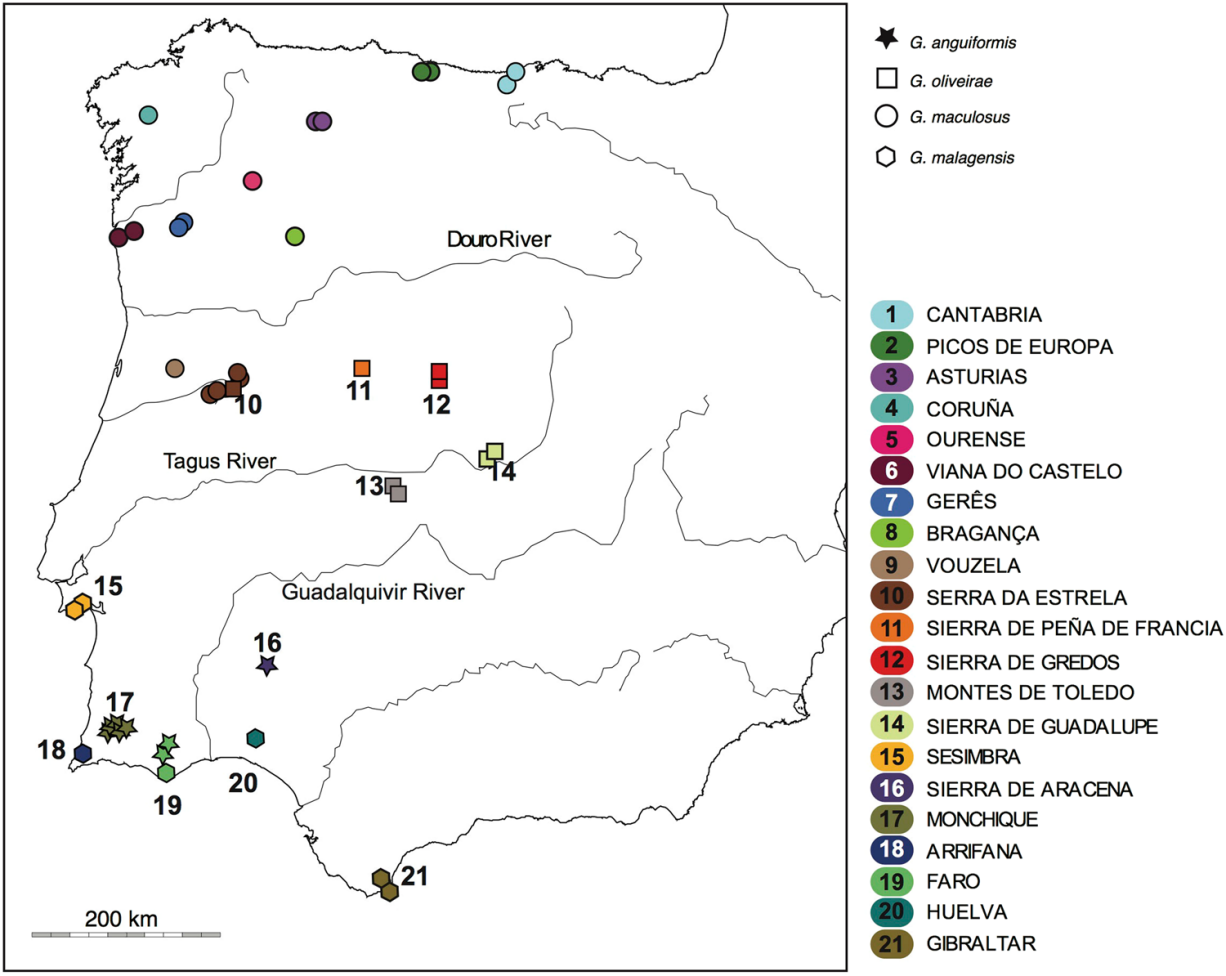
Map showing sample locations of *Geomalacus* (further details on Supplementary material S3). Figure generated using the *worldHires* (http://CRAN.R-project.org/package=mapdata) function implemented in R language (R Core Team (2015). R: A language and environment for statistical computing. R Foundation for Statistical Computing, Vienna, Austria. URL https://www.R-project.org/) (version 3.3.1) and edited in Adobe Illustrator CS6 (version 16.0.0) (http://www.adobe.com/products/illustrator.html).

Mountain ecosystems offer vertical environmental gradients and frequently harbour higher levels of endemism than adjacent lowland areas^5^, particularly in species with reduced tolerance to desiccation and low dispersal abilities. In southern Europe, the Iberian Peninsula shows a mountain range orientation (east to west) that offers a wide range of climates (Alpine, Atlantic, Mediterranean and Desert) that promote speciation. The Iberian Peninsula shows an exceptionally high level of endemism among arionid slugs^6^. The genus *Arion* includes species with a Palearctic distribution throughout Europe with currently no less than 17 morphologically-defined, Iberian endemic species^7–10^. In contrast, the genus *Geomalacus* is endemic to the Iberian Peninsula and only four species are currently recognized viz. *G. oliveirae*, *G. anguiformis*, *G. malagensis* and *G. maculosus* ^11–14^ (Fig. 1). *Geomalacus maculosus* also occurs in Ireland but its presence is considered of anthropogenic origin^15^; therefore, its status as an Iberian endemic is retained in the present study. No other species of *Geomalacus* are found outside Iberia. The 17 Iberian endemic *Arion* represent about 53% of the European diversity; 32 species currently listed^9,16^ but this number is most likely underestimated.

The taxonomy of arionids is based on plastic characters such as body size and colour that in part may depend on diet, environment, and age^17^. Other characters include the anatomy of the reproductive apparatus, the interpretation of which is often difficult if not impossible in immature stages, often preventing the correct identification of juveniles^18^. While for the genus *Arion* there is a molecular phylogeny based on mitochondrial (NADH1) and nuclear (ITS1) sequence data^7,19^, the only molecular analyses of *Geomalacus* are focused on *G. maculosus* ^15^ or *G. malagensis* ^20^, with no data available for the remaining species within the genus.

In this study, we explored the following hypotheses to explain the differences in endemic species richness between two slug genera, *Geomalacus* and *Arion*: (1) cryptic diversity within *Geomalacus*; (2) differences in the crown group age of each genus; (3) distinct patterns of diversification rates (e.g. higher initial speciation rates in *Arion*), and (4) some combination of the above factors (e.g. an older clade with higher initial speciation rates). To do so, we performed tests to delimit species within *Geomalacus* using mitochondrial cytochrome oxidase subunit I (COI) and nuclear ribosomal small subunit (18 S rRNA) sequence data to assure the exact number of species within the clade. To estimate the crown group ages of both genera we included available COI sequences of Iberian endemic and non-endemic *Arion*. We analysed diversification rates over time for each genus, also testing which model of diversification fitted best the data.

## Results

### Species delimitation, phylogeny and dating

ABGD identified eight putative species within Iberian endemic *Geomalacus*: *G. maculosus, G. anguiformis, G. malagensis* and five within *G. oliveirae*. The spedeSTEM analysis (theta = 0.08) supported the existence of eight putative species within Iberian endemic *Geomalacus* (ln L = −10380.97, model likelihood = 1.0; Supplementary Table S1), the same as recovered by ABGD. Values for single and multiple AIC calculations are identical.

Within-group estimates of average divergence within *Geomalacus* based on COI data ranged between 0% (*G. oliveirae* - Peña de Francia) and 6.8% (*G. maculosus*) Net divergence between groups ranged from 11.4% (between *G. oliveirae* - Peña de Francia and *G. oliveirae* - Gredos) to 20.6% (between *G. maculosus* and *G. oliveirae* - Montes de Toledo) (Table 1).

**Table 1:** Pairwise uncorrected COI sequence divergence among *Geomalacus* (mean ± s.d.).

	*G. anguiformis*	*G. maculosus*	*G. malagensis*	*Geomalacus oliveirae*				
				Montes de Toledo	S. Gredos	S. Guadalupe	S. Peña de Francia	S. Estrela
*G. anguiformis*	0.040 ± 0.005							
*G. maculosus*	0.177 ± 0.013	0.068 ± 0.006						
*G. malagensis*	0.173 ± 0.014	0.156 ± 0.013	0.009 ± 0.002					
*G. oliveirae* Montes de Toledo	0.172 ± 0.013	0.206 ± 0.014	0.202 ± 0.015	0.044 ± 0.005				
*G. oliveirae* Sierra de Gredos	0.141 ± 0.012	0.176 ± 0.013	0.167 ± 0.015	0.170 ± 0.014	0.001 ± 0.0007			
*G. oliveirae* Sierra de Guadalupe	0.130 ± 0.012	0.161 ± 0.012	0.166 ± 0.014	0.160 ± 0.014	0.145 ± 0.013	0.010 ± 0.002		
*G. oliveirae* Peña de Francia	0.146 ± 0.013	0.176 ± 0.013	0.171 ± 0.014	0.157 ± 0.014	0.114 ± 0.012	0.137 ± 0.013	0.000	
*G. oliveirae* Serra da Estrela	0.143 ± 0.012	0.170 ± 0.013	0.180 ± 0.014	0.168 ± 0.013	0.128 ± 0.013	0.119 ± 0.013	0.121 ± 0.013	0.003 ± 0.002

**Figure 2:**
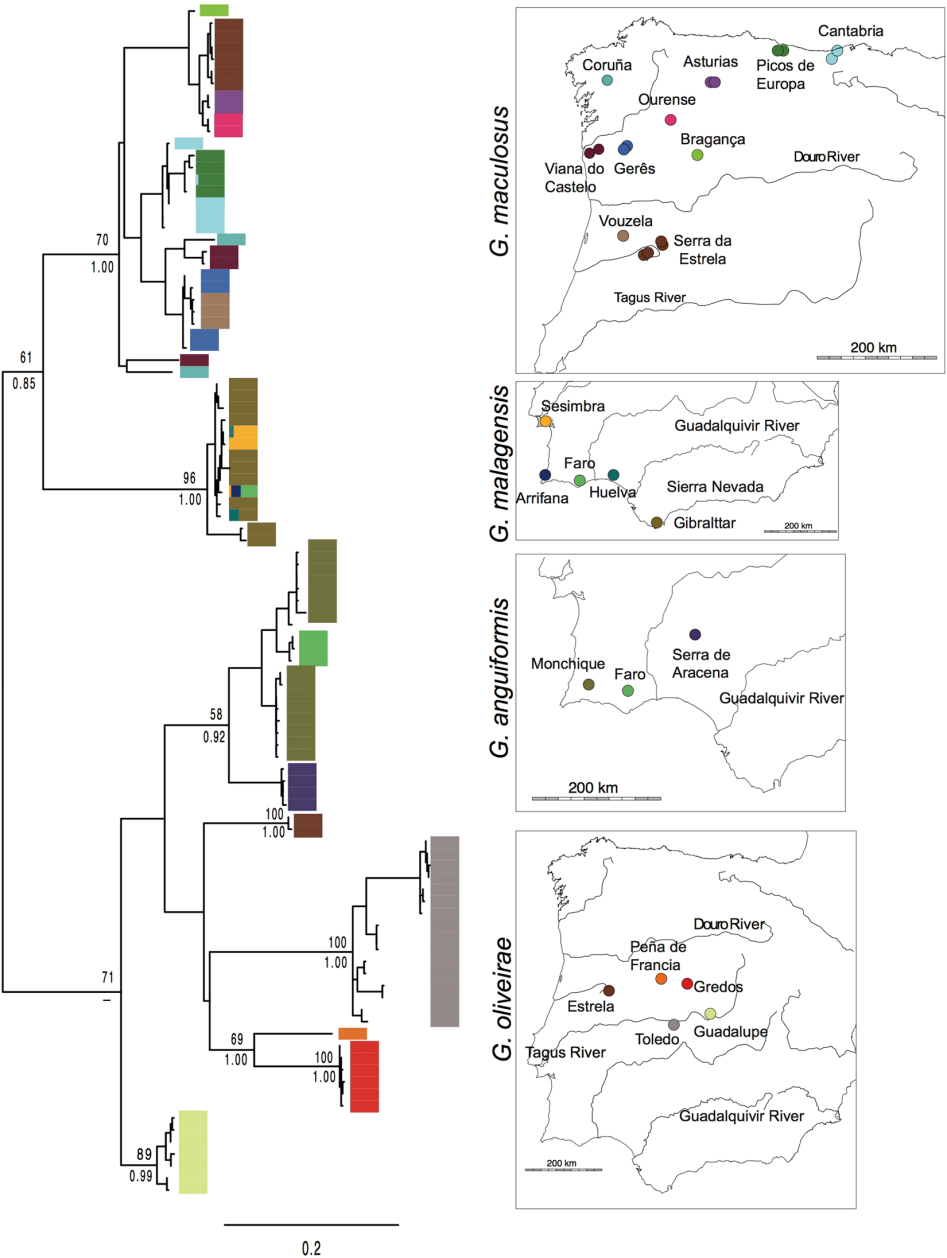
Phylogenetic relationships of *Geomalacus* based on a maximum likelihood analysis of 100 unique COI haplotypes from 21 sampling sites. Tip labels are haplotype codes and the number of sequences of each haplotype. Numbers at the nodes represent ML bootstrap values (top) and Bayesian posterior probabilities (bottom) for major clades. Colour bars indicate geographic origins. Maps indicate sample locations for each *Geomalacus* species and the corresponding colour for the geographic origin of each sample. Phylogenetic tree generated by FigTree http://tree.bio.ed.ac.uk/software/figtree and edited in Adobe Illustrator CS6 (version 16.0.0) (http://www.adobe.com/products/illustrator.html). Maps generated using the *worldHires* (http://CRAN.R-project.org/package=mapdata) function implemented in R language (R Core Team (2015). R: A language and environment for statistical computing. R Foundation for Statistical Computing, Vienna, Austria. URL https://www.R-project.org/) (version 3.3.1) and edited in Adobe Illustrator CS6 (version 16.0.0) (http://www.adobe.com/products/illustrator.html).

The maximum likelihood phylogeny based on the *Geomalacus* COI data set revealed five well-supported clades within the nominal species *Geomalacus oliveirae* that correspond to the species defined by both ABGD and spedeSTEM species delimitation methods (Fig. 2). *G. oliveirae* is recovered as paraphyletic due to the inclusion of *G. anguiformis* (Fig. 2). The BI topology was similar to the ML tree, except that includes all five *G. oliveirae* lineages on a single clade albeit with relatively low statistical support (BPP: 0.7; Fig. 2). BI analyses of the combined data set yielded similar results to the BI COI-based topology (Supplementary Figure S1).

The BI tree depicting the phylogenetic relationships within Arionidae based on COI sequence data is shown in the Supplementary Figure S2. While there is a single clade of *Geomalacus*, the 26 species of *Arion* grouped in two main clades that did not cluster together. One of two subclades of *Arion* (clade I; Supplementary Figure S2) is sister to *Geomalacus*. Iberian endemic *Arion* are not monophyletic as clade I included all Iberian endemic *Arion* plus six non-endemic species. The other clade of *Arion* (clade II) comprised non-endemic species only.

**Figure 3:**
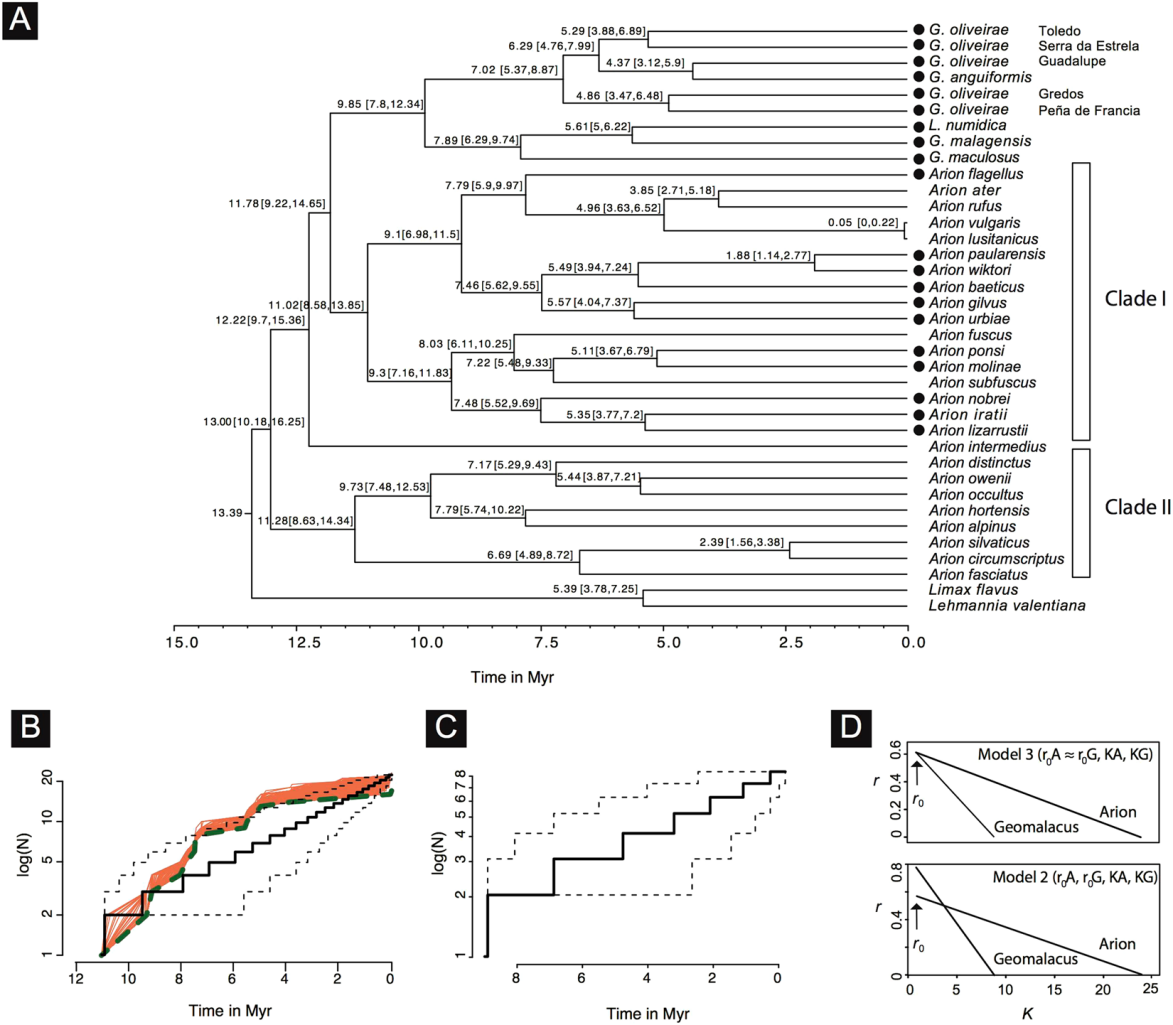
(**A**) Beast maximum clade credibility chronogram showing main cladogenetic events within two genera of terrestrial slugs (*Arion* and *Geomalacus*) based on a fragment of the mitochondrial COI gene. Black circles represent Iberian endemic species. Clade I includes all 11 Iberian endemic *Arion* plus six non-endemic *Arion* species whereas clade II includes eight non-endemic Iberian *Arion* species. Age estimates in million years and corresponding 95% highest posterior density intervals (values in square brackets) are depicted; (**B**) Lineage through time plots (LTT) obtained from the empirical Beast timetree after pruning clade II that included non-endemic *Arion* only. Solid line represents the LTT plot. Orange lines represent the LTT plots from 1000 simulations using the CorSim approach taking into account missing species. Dashed green line represents the mean LTT plot and the area enclosed by stippled lines indicated the 95% CI for 1000 trees simulated under a pure-birth model; (**C**) Lineage through time plots (LTT) from the empirical Beast timetree including *Geomalacus* only. Solid line represents the LTT plot. The area enclosed by stippled lines indicated the 95% CI for 1000 trees simulated under a pure-birth mode. No CorSim simulations were performed because the genus was completely sampled; (**D**) Net diversification rate (*r*) vs. carrying-capacity (*K*) plots from the best-fit diversity dependent model on top (*r*_0_A ≈ *r*_0_G, *K*A, *K*G) and the second best-fit model on bottom (*r*_0_A, *r*_0_G, *K*A, *K*G). Tree figure generated by FigTree http://tree.bio.ed.ac.uk/software/figtree, LTT plots and *r* vs *K* produced in R (https://cran.r-project.org/ and edited in Adobe Illustrator CS6 (version 16.0.0) (http://www.adobe.com/products/illustrator.html).

The estimated crown group ages of each clade recovered in the Beast analysis are the following: *Geomalacus* −9.85 [95% HPD: 7.8–12.34] myr; *Arion* clade I - 11.02 [95% HPD: 8.58–13.85] myr; *Arion* clade II - 11.28 [95% HPD: 8.63–14.34] myr (Fig. 3A).

### Macroevolutionary dynamics

The convex shape of the semi-logarithmic LTT plots derived from the *Arion* and *Geomalacus* trees (Fig. 3B and C) suggest a non-constant diversification rate. The γ-statistic for the *Arion* tree differed significantly from a model of constant rate diversification (γ = −2.99, *p* < 0.001). Moreover, to discard the influence of incomplete sampling due to the existence of *Arion* species not included in the phylogeny, we used the γ-statistic to simulate a null distribution of γ (assuming a constant rate pure-birth process) with 1000 replicates and assuming an incompletely sampled phylogeny with a possible total number of 25, 35 and 40 *Arion* species. The simulation returned a γ-critical value of −2.05 (*p* = 0.005) for 25 missing species, −2.31 (*p* = 0.010) for 35 missing species, and −2.47 (*p* = 0.015) for 40 missing species. The endemic *Geomalacus* phylogeny, including *Letourneuxia numidica*, and with no missing species, yielded a γ-value of −2.38 (*p* = 0.009). Therefore, all results rejected the hypothesis of a constant rate of diversification with *p* < 0.05, indicating that the negative γ values are significant when compared with the null model of the constant diversification rate, irrespective of the effect of taxon sampling.

To assess if diversification within the two clades of arionids (*Geomalacus* and the clade corresponding to all Iberian endemic *Arion* plus six non-endemic species, clade I) conformed with a constant rate or has changed over time, we used BAMM and maximum likelihood to fit the branching times derived from our dated tree to a variety of lineage diversification models. Bamm analyses using the ultrametric tree obtained with Beast showed no significant rate shifts (Supplementary Figure S3). Shift posterior distributions for 901 analysed samples (after burn-in) were the following: shift posterior distributions: zero shifts, *p* = 0.83; one shift, *p* = 0.13; two shifts, *p* = 0.03; three shifts, *p* = 0.004; four shifts, *p* = 0.002. The model that fitted best the data was the pure-birth constrained model (*λ Arion* = *λ Geomalacus*) (Table 2). To test for the potential effects of diversity-dependence, we compared the fit of pure-birth, birth-death with linear diversity-dependent (DDL), exponential diversity-dependent (DDX) and yule-2-rate models. We tested these models first, which do not account for missing species, as a preliminary test for diversity-dependence. Results showed that DDL was the model that fitted best for both *Arion* and *Geomalacus* clades (Tables 3 and 4). Furthermore, the diversity-dependent diversification model (DDD) is the model that fitted best, when including incomplete taxon sampling in *Arion* diversification (Table 5).

**Table 2:** Diversification tests using genus specific speciation and extinction rate-constant models.

Package	Function	IST	Model	*Arion (A)*		*Geomalacus (G)*		AIC	Δ AIC
				*λ*	*μ*	*λ*	*μ*		
diversitree	make.bd.split	yes	Pure-birth (*λ*A, *λ*G)	0.146	‡	0.433	‡	30.458	1.800
**diversitree**	**make.bd.split**	**yes**	**Constrained pure-birth** ***(λA = λ*** **G)**	**0.144**	‡	**0.144**	‡	**28.460**	**0.000**
diversitree	make.bd.split	yes	Birth-death (*λ*A, *λ*G, *μ*A, *μ*G)	0.146	0.000	0.143	0.000	34.458	5.810

Bold  identifies the best-fit model. AIC’s and the difference in AIC’s with the best model (∆AIC) are shown. IST, incomplete sampling taxa; *λ*A, the initial speciation rate in *Arion*; *λ*G, the initial speciation rate in *Geomalacus; μ*_0_A, the initial extinction rate in *Arion*; *μ*_0_G, the initial extinction rate in *Geomalacus*; *λ*, the initial speciation rate; *μ*, the initial extinction rate; ‡ in the pure-birth model, extinction is fixed to zero.

**Table 3:** *Arion* diversification tests using speciation and extinction rate-constant models and rate-variable linear diversity-dependent logistic (DDL), exponential diversity-dependent (DDX) and yule-2-rate.

Package	Function	IST	Model	*λ*_0_ or *r*_0_	*μ*_0_	Extra parameters	AIC	Δ AIC
Laser	fitdAICrc	no*	Pure-birth (*λ*A)	0.135	‡		30.666	7.525
Laser	fitdAICrc	no*	Birth-death (*λ*_0_A, *μ*_0_A)	0.135	0.000		32.666	9.525
**Laser**	**fitdAICrc**	no*	**DDL (** ***r***_***0***_ **A,** ***K*** **A)**	**1.579**	**‡**	***K*** **=** **16.727**	**18.831**	**0.000**
Laser	fitdAICrc	no*	DDX (*r*_0_A, *xp*A)	0.584	‡	*xp* = 1. 072	20.018	4.770
Laser	fitdAICrc	no*	yule2rate (*r*_0_A, *r*_1_A)	0.254	‡	*r*_1_ = 0.034, *t*1 = 3.85	22.658	3.827

Bold  identifies the best-fit model. AIC’s and the difference in AIC’s with the best model (∆AIC) are shown. IST, incomplete sampling taxa; *λ*_0_A, the initial speciation rate in *Arion*; *μ*_0_A, the initial extinction rate in *Arion*; *μ*_0_, extinction rate when applicable; *K*, carrying capacity parameter of DDL and DDD models; *r*_0_ (= *λ*_0_ − μ_0_), initial net diversification rate for diversity dependent linear (DDL), diversity dependent exponential (DDX), yule2rate (or constant net diversification rate for pure-birth and birth-death model) and DDD models; *r*1 = net diversification rate after the first shift at time *t*1; xp, exponent of DDX model; * package function does not include provision to take into account incomplete taxon sampling; ‡ the parameter in the model is fixed to zero.

**Table 4:** *Geomalacus* diversification tests using speciation and extinction rate-constant models and rate-variable linear diversity-dependent logistic (DDL), exponential diversity-dependent (DDX) and yule-2-rate.

Package	Function	IST	Model	*λ*_0_ or *r*_0_	*μ*_0_	Extra parameters	AIC	Δ AIC
Laser	fitdAICrc	no	Pure-birth (*λ*G)	0.115	‡		25.126	6.966
Laser	fitdAICrc	no	Birth-death (*λ*_0_G, *μ*_0_G)	0.115	0.000		27.126	8.966
**Laser**	**fitdAICrc**	**no**	**DDL (** ***r***_***0***_ **G,** ***K*** **G)**	**1.807**	**‡**	***k*** **=** **8.088**	**9.044**	**0.000**
Laser	fitdAICrc	no	DDX (*r*_0_G, *xp*G)	1.358	‡	*xp* = 1.407	22.345	5.758
Laser	fitdAICrc	no	yule2rate (*r*_0G_, *r*_1_G)	0.337	‡	r_1_ = 0.043, t1 = 5.29	22.229	7.599

Bold  identifies the best-fit model. AIC’s and the difference in AIC’s with the best model (∆AIC) are shown.IST, incomplete sampling taxa; *λ*_0_G, the initial speciation rate in *Geomalacus*; *μ*_0_G, the initial extinction rate in *Geomalacus*; *μ*_0_, extinction rate when applicable; *K*, carrying capacity parameter of DDL and DDD models; *r*_0_ (=*λ*_0_ − μ_0_), initial net diversification rate for diversity dependent linear (DDL), diversity dependent exponential (DDX), yule2rate (or constant net diversification rate for pure-birth and birth-death model) and DDD models; *r*1 = net diversification rate after the first shift at time *t*1; xp, exponent of DDX model; * package function does not include provision to take into account incomplete taxon sampling, but *Geomalacus* has a complete sampling; ‡ the parameter in the model is fixed to zero.

**Table 5:** *Arion* diversification tests using rate-constant (pure-birth, birth-death) and diversity-dependent diversification decoupled model (DDD), yule-2-rate and time-varying speciation and constant extinction (SPVAR).

Package	Function	IST	Model	*λ*_0_ or *r*_0†_	Extra parameters	*μ*_0_	*K*	AIC	Δ AIC
DDD	dd_ML	yes	Birth-death (*λ*_0_A, *μ*_0_A)	0.189		0.000	Inf	31.562	11.41
**DDD**	**dd_ML**	**yes**	**DDD (** ***r***_***0***_ **A,** ***K*** **A)**	**0.586**		**‡**	**23.046**	20.147	**0.00**
diversitree	make.bd.t	yes	yule2rate (*r*_0_A, *r*_1_A)	0.040	*r*_1_ = 0.285	‡	‡	23.586	3.44
diversitree	make.bd.t	yes	SPVAR (*r*_0_A, *r*_1_A, *k*A)	0.046	*k* = −0.248	0.000	‡	24.278	4.13

Bold  identifies the best-fit model. AIC’s and the difference in AIC’s with the best model (∆AIC) are shown. IST, incomplete sampling taxa; *λ*_0_A, the initial speciation rate in *Arion*; *μ*_0_A, the initial extinction rate in *Arion*; *μ*_0_, extinction rate when applicable; *K*A, carrying capacity parameter of DDD model; *r*_0_ (= *λ*_0_ − μ_0_), initial net diversification rate for DDD, yule2rate andSPVAR models; *r*_1_ = net diversification rate after the first shift at time *t*_1_; *k*, parameter of the exponential change in speciation rate for the models SPVAR; † in this study μ_0_ is zero therefore *r*_0_ is effectively equal to *λ*_0_. ‡ the parameter in the model is fixed to zero.

Given the high support obtained for diversity-dependent models against the null hypothesis of diversity independent speciation rate (with or without sampling fraction), we evaluated six linear diversity-dependent, null extinction models accounting for incomplete sampling. The best-supported model was model 3 (Table 6) with diversity-dependent cladogenesis, identical initial diversification rates for both clades (*r*_0_A = *r*_0_G), and independently estimated carrying capacities (*K*) for both clades. The second-best model (∆ AIC = 1.46) indicates diversity-dependent cladogenesis, different initial diversification rates for both clades (*r*_0_A ≠ *r*_0_G), and independently estimated carrying capacities (*K*) for both clades. Models 4 to 6 with clade-dependent *K* values received substantially less support (∆ AIC > 20) than models with genera-independent *K*’s (∆ AIC < 6). To account for a larger number of missing taxa, we have performed all estimations increasing the missing species to 23, yielding a total of 40 *Arion* species. The best performing model is also model 3 (Supplementary Table S2).

**Table 6:** Diversification test using diversity-dependent diversification decoupled (DDD) models using genus specific initial net diversification rate, initial extinction rate and carrying capacity parameter.

Package	Function	IST	Model	***Arion***			***Geomalacus***			AIC	Δ AIC
				*r*_0_	*μ*_0_	*K*	*r*_0_	*μ*_0_	*K*		
DDD	dd_KI_ML	yes	*r*_0_A, *r*_0_G, *μ*_0_A, *μ*_0_G, *K*A, *K*G	0.61	0.021	22.982	0.823	2.00E-05	9.009	129.72	4.88
DDD	dd_KI_ML	yes	*r*_0_A, *r*_0_G, *K*A, *K*G	0.538	‡	23.217	0.828	‡	8.988	125.93	1.08
**DDD**	**dd_KI_ML**	**yes**	***r***_**0**_ **A** ** ≈** *r*_**0**_ **G,** ***K*** **A,** ***K*** **G**	**0.616**	‡	**22.952**	**0.616**	**‡**	**9.003**	**124.84**	**0**.**00**
DDD	dd_KI_ML	yes	*r*_0_A, *r*_0_G, *K*A ≈ KG	0.229	‡	56.12	1.902	‡	56.12	186.47	61.62
DDD	dd_KI_ML	yes	*r*_0_A, *r*_0_G, *K*A ≈ Inf, *K*G ≈ Inf	0.169	‡	Inf	0.125	‡	Inf	148.72	23.87
DDD	dd_KI_ML	yes	*r*_0_A ≈ *r*_0_G, *K*A ≈ Inf, *K*G ≈ Inf	0.154	‡	Inf	0.154	‡	Inf	147.17	22.33

Bold identifies the best-fit model. AIC’s and the difference in AIC’s with the best model (∆AIC) are shown. IST, incomplete sampling taxa; *r*_0_A (=*λ*_0_A − μ_0_A), initial net diversification rate for *Arion*; *r*_0_G (=*λ*_0_G − μ_0_G), initial net diversification rate for *Geomalacus*; *μ*_0_A, the initial extinction rate in *Arion*; *μ*_0_G, the initial extinction rate in *Geomalacus*; *μ*_0_, the initial extinction rate when applicable; *K*A, carrying capacity parameter for *Arion*; *K*G, carrying capacity parameter for *Geomalacus*; *r*_0_ (= *λ*_0_ − μ_0_), initial net diversification rate; ‡ the parameter in the model is fixed to zero.

## Discussion

Current information about species richness of Iberian endemic terrestrial slugs suggests an apparent striking disparity between the two sister clades, *Geomalacus* and *Arion* clade I that includes all 11 Iberian endemics plus six non-endemic species. While 17 Iberian endemic species are listed within *Arion*, only four species are reported for *Geomalacus*. Establishing the number of Iberian endemic *Arion* was not trivial because species classification is largely based on morphology that is most probably affected by the existence of uncovered diversity and possibly conspecifics, not evaluated in the present work. Given these caveats, we judge the 17 Iberian endemic species to be a balanced list, subject however to future changes. Our species delimitation tests indicated the existence of five, morphologically similar, putative species within *Geomalacus oliveirae*, which raises to eight the number of lineages within the genus. Seven out of the eight putative *Geomalacus* species show a distribution predominantly associated with mountain regions. *Geomalacus anguiformis* is reported only from mountain areas of southern Iberia (Caldeirão, Monchique and Aracena), and each of the five lineages recovered within *G. oliveirae* are restricted to a single mountain area in central Iberia (Serra da Estrela, Peña de Francia, Gredos, Guadalupe or Montes de Toledo). Mountain areas have long been recognized as biodiversity hotspots due to their ecological heterogeneity^21^. For instance, a recent study showed a radiation of freshwater gastropods inhabiting the Iberian mountain ranges strongly connected to the geographic evolution of river basins^22^. Our results suggest that *G. oliveirae* has undergone an allopatric speciation process most likely driven by fragmentation of the suitable habitat that restricted its distribution to mountain areas. Species with fragmented distributions typically show reduced gene flow among populations^23^, which may promote speciation. We did not detect cryptic diversity within *G*. *anguiformis* but the relatively deep divergence between specimens of Aracena (Fig. 2) and the remaining mountain locations suggests that this species may be undergoing a speciation process.

The disparity between the Iberian endemic arionids is lessened if the cryptic diversity within *Geomalacus* is interpreted to represent valid species; nevertheless, the differences are still significant (*Arion*: 17 species *vs*. *Geomalacus*: 8 species) and other factors must be exerting control on species diversity. Although there are no quantitative observations on their ecological behaviour, anecdotal observations (C. Patrão, pers. obsv.) revealed the existence of several characteristics of *Arion* slugs that may represent an evolutionary advantage: (1) are more generalist than *Geomalacus* in their feeding; (2) some *Arion* species seem to disperse faster; (3) have wider environmental tolerance (unlike *Geomalacus*, *Arion* may survive in areas with no running water), and (4) show a wider geographic distribution. Ecological niche modelling further supports the narrower environmental tolerance of *Geomalacus* ^14^.

It is a reasonable expectation to assume that in extant clades higher species richness should correlate with clade age because older clades had more time to accumulate species^24^. Nevertheless, the three clades of arionid slugs retrieved in the dating analysis did not show differences in their crown-group ages that justify such disproportion in endemic species richness (*Geomalacus*: 9.85 myr; *Arion* clade I that includes all Iberian endemics plus six non-endemic lineages: 11.02 myr; *Arion* clade II that comprises non-endemic species only: 11.28 myr).

Another hypothesis that could explain the observed differences could be distinct diversification rate dynamics^25^. BAMM indicated absence of rate shifts within the three clades and macroevolutionary analyses supported two equally plausible models (ΔAIC ≤ 2) both showing diversity-dependent diversification and null extinction within *Arion* (clade I) and *Geomalacus* that do not explain differences in species richness. The diversity-dependent models imply a decrease in the speciation rate towards the present, as the number of species increases^26^ and niche space becomes saturated. However, the two equally-supported models indicated variable carrying capacities for both genera and a weaker rate decline for *Arion* that may provide an explanation for the higher number of species shown by this genus. The only difference between the two equally-plausible models concerns the initial speciation rate; while model 2 shows higher initial rates for *Geomalacus* and a stronger rate decline throughout time, model 3 specifies similar initial speciation rates for both groups. Nevertheless, both models clearly justify the higher number of *Arion* (and/or the lower number of *Geomalacus* species).

The higher diversity originated in the early history of *Arion* and *Geomalacus* is most likely an important reason for the observed slowdown towards the present. The two genera may overlap in food requirements, but the broader environmental tolerance combined with a faster dispersal and wider distribution may have represented an evolutionary advantage for *Arion*. The fragmented geographic distribution of *Geomalacus* in the mountain areas might have promoted a higher initial speciation rate because absence of gene flow between isolated populations but as niche space becomes saturated, a stronger decline in speciation rates is expected, which is supported by model 2. The interspersed inclusion of all Iberian endemic *Arion* with other non-endemic species in clade I suggests multiple colonizations of the Iberia Peninsula that could ultimately lead to more *Arion* species (Fig. 3).

Differences in Iberian endemic species richness between two genera of terrestrial slugs were not caused by a significantly earlier origin of the more specious genus (*Arion*). Regardless the differences between the two equally plausible diversification models (similar initial speciation rates *vs*. higher initial speciation rates in *Geomalacus*), both coincide in a weaker rate decline in *Arion* and diversity-dependent diversification for both groups. A broader environmental tolerance combined with faster dispersal and wider distribution allowed multiple colonizations of the Iberian Peninsula that further explain the higher species richness of *Arion* in this geographical area.

## Methods

### Taxon sampling

For the present study, we have chosen species endemic from the Iberian Peninsula biogeographical province, including the Pyrenees endemics. The choice of *Geomalacus* species was straightforward, as apart from *G. maculosus*, which apparently was recently introduced in Ireland accidentally and human assisted^15^, the genus only exists in Iberia. A total of 492 specimens representing the four nominal *Geomalacus* species were collected from 49 localities between 2007 and 2010 (Fig. 1 and Supplementary Tables S3 and S5). *Geomalacus* was identified following Castillejo *et al*.^12^. Foot tissue was preserved in 96% ethanol and stored at −20 °C. After perusing the existing literature^7,27^, elected species within *Arion* were chosen. The list of species includes 17 *Arion* species (Electronic supplementary Table S4), for which molecular data only exists for 10 of those species. Additionally, the Moroccan species *Letourneuxia numidica* was included for the dating analysis (see section Dating analysis).

### DNA extraction, amplification and sequencing

Total genomic DNA of the 492 individuals was extracted with a chelex buffer using a modified protocol of Walsh *et al*.^28^: foot tissue was added to 250 µL of 5% (w/v) solution of chelex with 5 μL of Proteinase K, and incubated with thorough mixing at 55 °C for 60 min, followed by a 20 min incubation at 95 °C. The samples were then placed on ice for 2 min and centrifuged at 11,000 rpm for 3 min. The supernatant containing genomic DNA was removed and used directly as a template in downstream polymerase chain Reaction (PCR) analysis. Amplifications of a 750 bp fragment of the mitochondrial DNA COI gene were obtained using primers LCO1490 and HCO2198^29^. Additionally, for a subset of individuals representing the major evolutionary lineages inferred by species delimitation tests, a 700 bp-fragment of the small subunit nuclear ribosomal gene (18 S) was amplified and sequenced with the primers 4F18S and 1R18S^30^. PCR profiles were: one cycle of 5 min at 95 °C, 40 cycles of 40 s at 95 °C, 40 s at 40 °C and 1 min at 72 °C and a last elongation step of 5 min at 72 °C. PCR amplifications were performed in a total 25 µl reaction volume of 1X buffer, 2 mM MgCl2, 0.2 mM DNTP’s, 0.2 µM of each primer and 1U Taq DNA polymerase Promega (Madison, USA). Amplification products were purified by ethanol/sodium acetate precipitation and directly sequenced using the corresponding PCR primers. Sequencing was performed on an ABI 3130xl (Applied Biosystems) automated sequencer using the BigDye Deoxy Terminator cycle-sequencing kit (Applied Biosystems) following manufacturer’s instructions. All new sequences were deposited in GenBank (Supplementary Tables S3 and S5).

### Phylogenetic analyses

#### Geomalacus

DNA sequences were aligned with Mafft v. 7.245^31^ using the “—auto” option that automatically selects the appropriate strategy according to data size. Uncorrected, pairwise COI nucleotide diversities within and between the *Geomalacus* species were estimated in the software package MEGA 6.0.6 ^32^. Identical haplotypes were collapsed with DNACOLLAPSER (http://users-birc.au.dk/biopv/php/fabox/dnacollapser.php). Two data sets were used in all phylogenetic analyses: (1) COI data set including all unique Geomalacus haplotypes (100 sequences, 594 bp); (2) the combined data set that included only individuals sequenced both for COI and the nuclear 18 S fragment (74 sequences, COI: 594 bp and 18 S: 524 bp).

Bayesian Inference (BI) analyses based on the COI and the combined data sets were performed with MrBayes v. 3.2.1^33^. The BI analysis of the COI data set was analysed under the GTR + I + Γ, the best-fit model selected by Modeltest ^34^. BI analysis of the combined data set included two data partitions: COI and 18 S. Each partition was analysed according to the best-fit model selected by Modeltest (COI: GTR + I + Γ; 18 S: JC + I). Model and model parameters were estimated independently for each of the data partitions using the unlink command in MrBayes. Analyses accommodated among-partition rate variation through use of the “prset applyto = (all) ratepr = variable;” command in MrBayes. All analyses were run for 1 × 10^7^ generations (four simultaneous Markov chains; 1 × 10^3^ sample frequency) following a discarded burn-in of 10%. The convergence to the stationary distributions was confirmed by inspection of the MCMC samples using Tracer v. 1.6^35^. *Arion ater* was selected as outgroup.

Maximum Likelihood (ML) analyses based on the COI and the combined data sets were performed with RAxML v. 8.2.8^36^. COI-based analyses were run under the GTRGAMMA evolutionary model. Individual α-shape parameters, GTR-rates, and empirical base frequencies were estimated and optimized for each partition of the combined data set (COI and 18 S) used in this analysis. For both analyses, the best-scoring ML tree was determined from 100 randomized maximum-parsimony starting trees using the rapid hill-climbing algorithm and 1 × 10^3^ bootstrap replicates were drawn on the best-scored ML tree using the exhaustive bootstrap algorithm. *Arion ater* was selected as outgroup.

#### Arionidae

Bayesian Inference (BI) analysis was performed in Beast v1.8.4^37^ to analyse the phylogenetic relationships within the family Arionidae. We used COI sequences (42 taxa; 523 bp) that represent one individual per species of *Geomalacus* recovered by ABGD and spedeSTEM, 26 *Arion* species from which 11 are Iberian endemic, and all genera of Arionidae available in the GeneBank (accession numbers in Table S4). MCMC analyses were run for 100,000,000 generations with a sample frequency of 10,000, following a discarded burn-in of 10,000,000 steps. The convergence to the stationary distributions was confirmed by inspection of the MCMC samples using Tracer v1.6^35^.

#### Species delimitation in *Geomalacus*

Given the asymmetry in species richness between *Arion* and *Geomalacus* we suspected of the existence of cryptic diversity within the latter; therefore, we used ABGD (Automatic Barcode Gap Discovery)^38^ and the COI data set to delimit species within *Geomalacus*. ABGD is based on the identification of the barcode gap, i.e. a larger divergence among individuals of different species than among conspecific individuals. We selected the Jukes-Cantor model and the X - value of 0.9 (minimum relative barcoding gap width), and prior intraspecific divergences ranging from Pmin = 0.001 to Pmax = 0.12 to run the barcode gap analysis.

We also used spedeSTEM^39,40^ to further analyse the number of putative species within *Geomalacus*. This method calculates the maximum likelihood species tree from all hierarchical arrangements of the sampled alleles and uses information theory to quantify the model probability of each permutation. Specifically, the probabilities of multiple permutations of putative evolutionary lineages are calculated using STEM 2.0^41^. It returns a table of models ranked according to their probability. Ultrametric trees for each gene partition are required and a user-supplied estimate of θ. We used DnaSP v.5^42^ to compute the average theta for the two loci, and Beast2.4^43^ to infer both trees (separately) based on the COI and 18 S data sets. MCMC analyses were run for 1 × 10^8^ generations with a sample frequency of 10,000, following a discarded burn-in of 1 × 10^7^ steps. The convergence to the stationary distributions was confirmed by inspection of the MCMC samples using Tracer v1.6.

#### Evolutionary rates through time

To explore how the variation of diversification rates (speciation and extinction) affected the observed differences in species richness, we must evaluate three main hypotheses: (1) differences in the crown group age of each genera; (2) distinct patterns of diversification rates (e.g. higher initial speciation rates in *Arion* and/or diversity-dependence), and (3) a combination of the two (e.g. an older clade with higher initial and diversity dependence with weaker decline in diversification rates).

A Bayesian relaxed molecular-clock approach implemented in Beast v1.8.4^37^ addresses the first hypothesis by estimating the crown group ages of *Geomalacus* and *Arion*. To perform this analysis, we used COI sequences (37 taxa; 543 bp) from one individual per species of *Geomalacus* recovered by ABGD and spedeSTEM and COI sequences from all *Arion* species available in the GeneBank (accession numbers in Table S1). We also included the Moroccan species *Letourneuxia numidica* because estimates of divergence are usually based upon well-known historical events, geologic or fossil, which can be used as calibration points to estimate taxon-specific mutation rates^44^. Yet, as there are no reliable arionid fossils, we used the opening of the Strait of Gibraltar [5.96–5.35 million years (myr) ago^45^], to calibrate the divergence between *G. malagensis* and *L. numidica*. *Lehmannia valentiana* and *Limacus flavus*, two Stylommatophorans from the superfamily Limacoidea were selected as outgroups^46^.

After testing different tree (Yule and Birth-Death) and clock (uncorrelated lognormal and strict) priors we have chosen a Birth-Death Incomplete Sampling tree prior and placed a normal distribution in the divergence of *G. malagensis* and *L. numidica*, considered more appropriate when using biogeographic events to calibrate the tree^47^. Parameters were: mean in real space, M = 5.645 and standard deviation, S = 0.315. MCMC analyses were run for 100,000,000 generations with a sample frequency of 10,000, following a discarded burn-in of 10,000,000 steps. The convergence to the stationary distributions was confirmed by inspection of the MCMC samples using Tracer v1.6.

To address hypotheses (2) and (3) we used the ultrametric tree obtained from the Beast analysis from which the Moroccan species *Letourneuxia numidica* was pruned and only *Arion* clade I (includes all 11 Iberian endemic *Arion* plus six non-endemic *Arion* species) was considered because we were interested in analysing through time variation of the diversification rates of Iberian endemic arionids. We used the R package^48^ *laser* ^49^ to compute the γ-statistic^50^ and *phytools* ^51^ to construct the lineage through time (LTT) plots, for each clade. For complete phylogenies, γ = ±1.645 represents the critical value of the constant-rate test^50^. Values outside this interval reject the null hypothesis of the Yule pure-birth model (no extinction). This approach yields a null distribution of values against which the empirical γ is compared using the Monte Carlo constant rates (MCCR) test statistic^50^. However, the result of the “*gamStat”* function (*laser* package) does not take into account incomplete sampling. Therefore, we used the estimated γ-statistic and simulated 10,000 replicates to account for these missing taxa, using “*mccrTest”* function also available in *laser* ^49^. This procedure, randomly prunes the number of missing taxa from each replicated tree, to simulate incomplete taxa sampling. A null distribution of the γ-statistic is generated from these replicates, and the observed γ-statistic from the previous test is compared to this distribution.

We used BAMM-Bayesian analysis of Macroevolutionary Mixture^52^ to detect heterogeneity in speciation rates across the time-calibrated tree obtained with Beast while accounting for non-random incomplete sampling (we used useGlobalSamplingProbability = 0 and then set the corresponding sampling fractions in the file sample_probs.txt). We set four rj-MCMC runs for 200 × 10^6^ generations sampled every 200 × 10^3^ generations. We used the function “*setBAMMpriors*” in R to select more appropriate prior values and ESS (effective sample size) to assess the convergence of the runs and considered values above 200 as indicating convergence^53^.

Additionally, to handle with incomplete taxon sampling in *Arion* clade I, we applied the “*CorSiM”* (“Correction by Simulating Missing splits”) approach^54^ using the *TreeSim* R-package^55^ to simulate 1000 trees under a constant speciation and extinction rate. “*CorSiM”* requires as input data a speciation and extinction rates that we obtained from the best-fit lineage diversification model (see Results).

To further evaluate hypotheses (2) and (3), we compared constant genera-decoupled rate models, i.e., models that evaluate simultaneously separate parameters for each clade, to assess if constant speciation rates (*λ)*, and constant extinction rates (*μ)* are homogeneous: pure-birth (*λ Arion* ≠ *λ Geomalacus*), constrained pure-birth (*λ Arion* ≈ *λ Geomalacus*) and birth–death models (*λ, μ Arion* ≠ *λ, μ Geomalacus*)^56^ (see Supplementary Text S1 for details). Because the selected best-fit model based on these parameters (constrained pure birth, see Results and Box 1 from Supplementary information for further details) did not explain difference in species richness, we had to evaluate models that contemplate variable speciation rates such as diversity-dependence while assuming extinction rate to be zero: (1) linear diversity-dependent speciation rate (DDL) and exponential diversity-dependent declining speciation rates as a function of extant lineages (DDX)^49^; (2) single shift in speciation rate at a certain time (yule2rate); (3) diversity-dependent diversification model (DDD)^57^ and (4) time-varying speciation only (SPVAR)^56^ (see Supplementary Text S1 for details).

As the best-fit model indicates a diversity-dependent diversification (see Results and Box 1 from Supplementary information), we proceeded to evaluate clade decoupling models, i.e., models which evaluate simultaneously separate parameters for each genus. We used the R package *DDD* ^57^ that evaluates six different diversity-dependent models (see details in Supplementary Text S1). These models were tested using the following R-packages^48^: *laser* v2.4^49^ (function: “*fitdAICrc”*); *diversitree* V.0.9^56^ (functions: “*make.bd.split”*, “*make.bd.t”*) and DDD v.3.4^26,57,58^ (functions: “*dd_ML”*, “*dd_KI_ML”*). (see Supplementary Text S1 for details). The best supported models were those with low Akaike scores, and that deviate from the best model by less than two units (i.e., ∆AIC < 2^59^.

All analyses were performed on the R2C2 research group cluster facility, provided by the IT department of the University of Algarve.

## Electronic supplementary material

Supplementary_info
