# Supplementary material for: Different diversity-dependent declines in speciation rate unbalances species richness in terrestrial slugs
Source: Sci Rep. 2017 Nov 23;7:16198. doi: 10.1038/s41598-017-16417-y (PMC5700933; doi:10.1038/s41598-017-16417-y)

Different diversity-dependent declines in speciation rate unbalances species richness in terrestrial slugs

REGINA L. CUNHA, CLÁUDIA PATRÃO AND RITA CASTILHO

CCMAR (Centre of Marine Sciences) - Campus de Gambelas, Universidade do Algarve, 8005-139 Faro, Portugal.

Supplementary Table S1. SpedeSTEM results for single and multiple AIC calculations. The best model that supported an eight-species scenario within endemic *Geomalacus* is shown in bold.

Single AIC calculation

K	-ln	AIC	delta	model Likelihood	wi
1	16360.42	32722.85	9406.5	0	0
2	14092.81	28189.62	4873.34	0	0
3	13030.82	26067.64	2751.36	0	0
4	12185.57	24379.13	1062.85	0	0
5	11892.34	23794.68	478.4	0	0
6	11784.61	23581.23	264.94	0	0
7	11676.85	23367.69	51.41	6.8E-12	6.8E-12
8	11661.34	23338.68	22.4	1.4E-05	1.4E-05
**9***	**11694.14**	**23316.28**	**0**	**1**	**1**

Multiple AIC calculations

K	-ln	AIC	delta	model Likelihood	wi
1	16360.42	32722.85	9406.5	0	0
2	14092.81	28189.62	4873.34	0	0
3	13030.82	26067.64	2751.36	0	0
4	12185.57	24379.13	1062.85	0	0
5	11892.34	23794.68	478.4	0	0
6	11784.61	23581.23	264.94	0	0
7	11676.85	23367.69	51.41	6.8E-12	6.8E-12
8	11661.34	23338.68	22.4	1.4E-05	1.4E-05
**9***	**11694.14**	**23316.28**	**0**	**1**	**1**

* outgroup included

K = nº species

ln = likelihood of the species tree

wi = model probabilities, which measure the proportion of the total model likelihood (i.e., across all models) represented by that particular model (Carstens & Dewey, 2010)

Carstens BC, Dewey TA (2010) Species delimitation using a combined coalescent and information-theoretic approach: an example from North American Myotis bats. Syst Biol 59, 400-414.

Supplementary Table S2. Results for diversity-dependent diversification decoupled model (DDD) for *Geomalacus* (all species) and *Arion* total species = 40.

Package	Function	IST	Model	*Arion*			*Geomalacus*			AIC	Δ AIC
				r_0	μ_0	K	r_0	μ_0	K		
DDD	dd_KI_ML	yes	r_0A, r_0G, μ_0A, μ_0G, KA, KG	0,653	0,0459	51,79	0,781	0,0000	8,00	126,12	5,01
DDD	dd_KI_ML	yes	r_0A, r_0G, KA, KG	0,580	‡	52,50	0,779	‡	8,00	122,58	1,46
DDD	dd_KI_ML	yes	r_0A ≈ r_0G, KA, KG	0,588	‡	52,25	0,588	‡	8,00	121,12	0,00
DDD	dd_KI_ML	yes	r_0A, r_0G, KA ≈ KG	0,321	‡	2995,36	10,000	‡	2995,36	210,66	89,55
DDD	dd_KI_ML	yes	r_0A, r_0G, KA ≈ Inf, KG ≈ Inf	0,227	‡	Inf	4,804	‡	Inf	155,57	34,46
DDD	dd_KI_ML	yes	r_0A ≈ r_0G, KA ≈ Inf, KG ≈ Inf	0,214	‡	Inf	0,214	‡	Inf	141,55	20,44

A, refers to *Arion;* G referes to *Geomalacus;* IST, incomplete sampling taxa; μ0, the initial extinction rate when applicable; *K*, carrying capacity parameter; r0 (= λ0-μ0), initial net diversification rate; AIC, Akaike information criterion; ΔAIC, the difference in Akaike information criterion score from the best-fit model (shaded grey). ‡, extinction fixed to zero; Inf, infinity.

Supplementary Table S3. Collection ID, species name, accession number, haplotype number, number of sequences of cytochrome oxidase subunit I (COI), location code, region, latitude and longitude for *Geomalacus* specimens. List of Iberian endemic *Arion* species and GenBank accession numbers of available COI sequences.

Collection ID	Species	**COI** Genebank Accession Numbers	Haplotype number	Number of sequences	Location Code	Region	Latitude	Longitude
ANGMOa0147	*G. anguiformis*	KF781852	1		MOa	Monchique	37.307	-8.570
ANGMOa0319	*G. anguiformis*	KF781853	1		MOa	Monchique	37.307	-8.570
ANGMOa0321	*G. anguiformis*	KF781854	1		MOa	Monchique	37.307	-8.570
ANGMOa0329	*G. anguiformis*	KF781855	1		MOa	Monchique	37.307	-8.570
ANGMOa0316	*G. anguiformis*	KF781856	1		MOa	Monchique	37.307	-8.570
ANGMOa0326	*G. anguiformis*	KF781857	1		MOa	Monchique	37.307	-8.570
ANGMOa0327	*G. anguiformis*	KF781858	1		MOa	Monchique	37.307	-8.570
ANGMOa0330	*G. anguiformis*	KF781859	1		MOa	Monchique	37.307	-8.570
ANGMOa0322	*G. anguiformis*	KF781860	1	17	MOa	Monchique	37.307	-8.570
ANGMOa0323	*G. anguiformis*	KF781861	1		MOa	Monchique	37.307	-8.570
ANGMOa0324	*G. anguiformis*	KF781862	1		MOa	Monchique	37.307	-8.570
ANGMOa0328	*G. anguiformis*	KF781863	1		MOa	Monchique	37.307	-8.570
ANGMOa0318	*G. anguiformis*	KF781864	1		MOa	Monchique	37.307	-8.570
ANGMOa0320	*G. anguiformis*	KF781865	1		MOa	Monchique	37.307	-8.570
ANGMOa0317	*G. anguiformis*	KF781866	1		MOa	Monchique	37.307	-8.570
ANGMOb0260	*G. anguiformis*	KF781872	1		MOb	Monchique	37.305	-8.588
ANGMOb0254	*G. anguiformis*	KF781877	1		MOb	Monchique	37.305	-8.588
ANGMOb0261	*G. anguiformis*	KF781867	2		MOb	Monchique	37.305	-8.588
ANGMOb0266	*G. anguiformis*	KF781871	2	3	MOb	Monchique	37.305	-8.588
ANGMOb0262	*G. anguiformis*	KF781878	2		MOb	Monchique	37.305	-8.588
ANGMOb0264	*G. anguiformis*	KF781868	3		MOb	Monchique	37.305	-8.588
ANGMOb0257	*G. anguiformis*	KF781869	3		MOb	Monchique	37.305	-8.588
ANGMOb0263	*G. anguiformis*	KF781870	3		MOb	Monchique	37.305	-8.588
ANGMOb0268	*G. anguiformis*	KF781873	3		MOb	Monchique	37.305	-8.588
ANGMOb0265	*G. anguiformis*	KF781874	3		MOb	Monchique	37.305	-8.588
ANGMOb0267	*G. anguiformis*	KF781875	3		MOb	Monchique	37.305	-8.588
ANGMOc0331	*G. anguiformis*	KF781879	3		MOc	Monchique	37.320	-8.536
ANGMOc0333	*G. anguiformis*	KF781880	3		MOc	Monchique	37.320	-8.536
ANGMOc0332	*G. anguiformis*	KF781881	3		MOc	Monchique	37.320	-8.536
ANGMOc0334	*G. anguiformis*	KF781883	3		MOc	Monchique	37.320	-8.536
ANGMOd0337	*G. anguiformis*	KF781885	3		MOd	Monchique	37.342	-8.488
ANGMOd0338	*G. anguiformis*	KF781886	3		MOd	Monchique	37.342	-8.488
ANGMOd1718	*G. anguiformis*	KF781887	3		MOd	Monchique	37.342	-8.488
ANGMOd0341	*G. anguiformis*	KF781889	3	28	MOd	Monchique	37.342	-8.488
ANGMOd0336	*G. anguiformis*	KF781890	3		MOd	Monchique	37.342	-8.488
ANGMOd0417	*G. anguiformis*	KF781891	3		MOd	Monchique	37.342	-8.488
ANGMOd0339	*G. anguiformis*	KF781892	3		MOd	Monchique	37.342	-8.488
ANGMOe0532	*G. anguiformis*	KF781893	3		MOe	Monchique	37.313	-8.549
ANGMOe0531	*G. anguiformis*	KF781895	3		MOe	Monchique	37.313	-8.549
ANGMOe0533	*G. anguiformis*	KF781896	3		MOe	Monchique	37.313	-8.549
ANGMOe1147	*G. anguiformis*	KF781897	3		MOe	Monchique	37.313	-8.549
ANGMOe1149	*G. anguiformis*	KF781898	3		MOe	Monchique	37.313	-8.549
ANGMOe0607	*G. anguiformis*	KF781899	3		MOe	Monchique	37.313	-8.549
ANGMOe0530	*G. anguiformis*	KF781900	3		MOe	Monchique	37.313	-8.549
ANGMOe0526	*G. anguiformis*	KF781903	3		MOe	Monchique	37.313	-8.549
ANGMOe0608	*G. anguiformis*	KF781904	3		MOe	Monchique	37.313	-8.549
ANGMOe0528	*G. anguiformis*	KF781905	3		MOe	Monchique	37.313	-8.549
ANGMOe0534	*G. anguiformis*	KF781906	3		MOe	Monchique	37.313	-8.549
ANGMOb0256	*G. anguiformis*	KF781876	4	1	MOb	Monchique	37.305	-8.588
ANGMOc0335	*G. anguiformis*	KF781882	5	1	MOc	Monchique	37.320	-8.536
ANGMOd0340	*G. anguiformis*	KF781884	6	1	MOd	Monchique	37.342	-8.488
ANGMOd0418	*G. anguiformis*	KF781888	7	2	MOd	Monchique	37.342	-8.488
ANGMOf0556	*G. anguiformis*	KF781915	7		MOf	Monchique	37.306	-8.499
ANGMOe0529	*G. anguiformis*	KF781894	8	1	MOe	Monchique	37.313	-8.549
ANGMOe0527	*G. anguiformis*	KF781901	9	1	MOe	Monchique	37.313	-8.549
ANGMOe0505	*G. anguiformis*	KF781902	10	1	MOe	Monchique	37.313	-8.549
ANGMOf0560	*G. anguiformis*	KF781907	11	2	MOf	Monchique	37.306	-8.499
ANGMOf0561	*G. anguiformis*	KF781912	11		MOf	Monchique	37.306	-8.499
ANGMOf0552	*G. anguiformis*	KF781908	12		MOf	Monchique	37.306	-8.499
ANGMOf0551	*G. anguiformis*	KF781911	12		MOf	Monchique	37.306	-8.499
ANGMOf0550	*G. anguiformis*	KF781917	12	5	MOf	Monchique	37.306	-8.499
ANGMOf0558	*G. anguiformis*	KF781918	12		MOf	Monchique	37.306	-8.499
ANGMOf0553	*G. anguiformis*	KF781919	12		MOf	Monchique	37.306	-8.499
ANGMOf0548	*G. anguiformis*	KF781909	13	2	MOf	Monchique	37.306	-8.499
ANGMOf0559	*G. anguiformis*	KF781910	13		MOf	Monchique	37.306	-8.499
ANGMOf0554	*G. anguiformis*	KF781913	14	2	MOf	Monchique	37.306	-8.499
ANGMOf0555	*G. anguiformis*	KF781916	14		MOf	Monchique	37.306	-8.499
ANGMOf0549	*G. anguiformis*	KF781914	15	2	MOf	Monchique	37.306	-8.499
ANGMOf0557	*G. anguiformis*	KF781920	15		MOf	Monchique	37.306	-8.499
ANGSBb1162	*G. anguiformis*	KF781921	16		SBb	Faro	37.273	-7.875
ANGSBb1863	*G. anguiformis*	KF781922	16		SBb	Faro	37.273	-7.875
ANGSBb1174	*G. anguiformis*	KF781923	16		SBb	Faro	37.273	-7.875
ANGSBb1864	*G. anguiformis*	KF781924	16		SBb	Faro	37.273	-7.875
ANGSBb1171	*G. anguiformis*	KF781925	16		SBb	Faro	37.273	-7.875
ANGSBb1170	*G. anguiformis*	KF781926	16		SBb	Faro	37.273	-7.875

Collection ID	Species	**COI** Genebank Accession Numbers	Haplotype number	Number of sequences	Location Code	Region	Latitude	Longitude
ANGSBb1158	*G. anguiformis*	KF781927	16	16	SBb	Faro	37.273	-7.875
ANGSBb1867	*G. anguiformis*	KF781928	16		SBb	Faro	37.273	-7.875
ANGSBb1175	*G. anguiformis*	KF781929	16		SBb	Faro	37.273	-7.875
ANGSBb1154	*G. anguiformis*	KF781932	16		SBb	Faro	37.273	-7.875
ANGSBb1160	*G. anguiformis*	KF781934	16		SBb	Faro	37.273	-7.875
ANGSBb1155	*G. anguiformis*	KF781935	16		SBb	Faro	37.273	-7.875
ANGSBb1163	*G. anguiformis*	KF781936	16		SBb	Faro	37.273	-7.875
ANGSBb1159	*G. anguiformis*	KF781939	16		SBb	Faro	37.273	-7.875
ANGSBb1176	*G. anguiformis*	KF781940	16		SBb	Faro	37.273	-7.875
ANGSBb1865	*G. anguiformis*	KF781941	16		SBb	Faro	37.273	-7.875
ANGSBb1153	*G. anguiformis*	KF781930	17	3	SBb	Faro	37.273	-7.875
ANGSBb1169	*G. anguiformis*	KF781933	17		SBb	Faro	37.273	-7.875
ANGSBb1866	*G. anguiformis*	KF781938	17		SBb	Faro	37.273	-7.875
ANGSBb1161	*G. anguiformis*	KF781931	18	2	SBb	Faro	37.273	-7.875
ANGSBb1172	*G. anguiformis*	KF781937	18		SBb	Faro	37.273	-7.875
ARAARA0525	*G. anguiformis*	KF781942	19	9	ARA	Aracena	37.878	-6.817
ARAARA0536	*G. anguiformis*	KF781944	19		ARA	Aracena	37.878	-6.817
ARAARA0538	*G. anguiformis*	KF781946	19		ARA	Aracena	37.878	-6.817
ARAARA0540	*G. anguiformis*	KF781948	19		ARA	Aracena	37.878	-6.817
ARAARA0542	*G. anguiformis*	KF781950	19		ARA	Aracena	37.878	-6.817
ARAARA0543	*G. anguiformis*	KF781951	19		ARA	Aracena	37.878	-6.817
ARAARA0544	*G. anguiformis*	KF781952	19		ARA	Aracena	37.878	-6.817
ARAARA0545	*G. anguiformis*	KF781953	19		ARA	Aracena	37.878	-6.817
ARAARA0546	*G. anguiformis*	KF781954	19		ARA	Aracena	37.878	-6.817
ARAARA0535	*G. anguiformis*	KF781943	20	3	ARA	Aracena	37.878	-6.817
ARAARA0537	*G. anguiformis*	KF781945	20		ARA	Aracena	37.878	-6.817
ARAARA0541	*G. anguiformis*	KF781949	20		ARA	Aracena	37.878	-6.817
ARAARA0539	*G. anguiformis*	KF781947	21	1	ARA	Aracena	37.878	-6.817
ARAARA0547	*G. anguiformis*	KF781955	22	1	ARA	Aracena	37.878	-6.817
LETTAa1851	*L. numidica*	KF806037	23	5	TAa	Morocco	35.784	-5.901
LETTAa1860	*L. numidica*	KF806046	23		TAa	Morocco	35.784	-5.901
LETTAa1857	*L. numidica*	KF806048	23		TAa	Morocco	35.784	-5.901
LETTAb1889	*L. numidica*	KF806049	23		TAb	Morocco	35.783	-5.851
LETTAb1890	*L. numidica*	KF806050	23		TAb	Morocco	35.783	-5.851
LETTAa1855	*L. numidica*	KF806038	24	1	TAa	Morocco	35.784	-5.901
LETTAa1850	*L. numidica*	KF806039	25	1	TAa	Morocco	35.784	-5.901
LETTAa1849	*L. numidica*	KF806040	26	2	TAa	Morocco	35.784	-5.901
LETTAa1848	*L. numidica*	KF806043	26		TAa	Morocco	35.784	-5.901
LETTAa1856	*L. numidica*	KF806041	27	4	TAa	Morocco	35.784	-5.901
LETTAa1854	*L. numidica*	KF806042	27		TAa	Morocco	35.784	-5.901
LETTAa1853	*L. numidica*	KF806044	27		TAa	Morocco	35.784	-5.901
LETTAa1847	*L. numidica*	KF806047	27		TAa	Morocco	35.784	-5.901
LETTAa1852	*L. numidica*	KF806045	28	1	TAa	Morocco	35.784	-5.901
LETLET0605	*L. numidica*		29	1	LET	Morocco	31.130	-7.900
LETLET0622	*L. numidica*		30	1	LET	Morocco	31.130	-7.900
LETLET0630	*L. numidica*		31	2	LET	Morocco	31.130	-7.900
LETLET0636	*L. numidica*		31		LET	Morocco	31.130	-7.900
LETLET0635	*L. numidica*		32	1	LET	Morocco	31.130	-7.900
MACBRA0203	*G. maculosus*	KF806051	33	15	BRA	Bragança	41.688	-6.751
MACBRA0204	*G. maculosus*	KF806052	33		BRA	Bragança	41.688	-6.751
MACBRA0205	*G. maculosus*	KF806053	33		BRA	Bragança	41.688	-6.751
MACBRA0206	*G. maculosus*	KF806054	33		BRA	Bragança	41.688	-6.751
MACBRA0207	*G. maculosus*	KF806055	33		BRA	Bragança	41.688	-6.751
MACBRA0208	*G. maculosus*	KF806056	33		BRA	Bragança	41.688	-6.751
MACBRA0209	*G. maculosus*	KF806057	33		BRA	Bragança	41.688	-6.751
MACBRA0210	*G. maculosus*	KF806058	33		BRA	Bragança	41.688	-6.751
MACBRA0211	*G. maculosus*	KF806059	33		BRA	Bragança	41.688	-6.751
MACBRA0212	*G. maculosus*	KF806060	33		BRA	Bragança	41.688	-6.751
MACBRA0213	*G. maculosus*	KF806061	33		BRA	Bragança	41.688	-6.751
MACBRA0214	*G. maculosus*	KF806062	33		BRA	Bragança	41.688	-6.751
MACBRA0215	*G. maculosus*	KF806063	33		BRA	Bragança	41.688	-6.751
MACBRA0216	*G. maculosus*	KF806064	33		BRA	Bragança	41.688	-6.751
MACBRA0492	*G. maculosus*	KF806065	33		BRA	Bragança	41.688	-6.751
MACCAa0922	*G. maculosus*	KF806066	34	7	CAa	Asturias	42.949	-6.395
MACCAa0923	*G. maculosus*	KF806067	34		CAa	Asturias	42.949	-6.395
MACCAa0924	*G. maculosus*	KF806068	34		CAa	Asturias	42.949	-6.395
MACCAa0925	*G. maculosus*	KF806069	34		CAa	Asturias	42.949	-6.395
MACCAa0926	*G. maculosus*	KF806070	34		CAa	Asturias	42.949	-6.395
MACCAa0927	*G. maculosus*	KF806071	34		CAa	Asturias	42.949	-6.395
MACCAa0928	*G. maculosus*	KF806072	34		CAa	Asturias	42.949	-6.395
MACCAb0806	*G. maculosus*	KF806073	35	8	CAb	Asturias	42.955	-6.372
MACCAb0807	*G. maculosus*	KF806074	35		CAb	Asturias	42.955	-6.372
MACCAb0808	*G. maculosus*	KF806075	35		CAb	Asturias	42.955	-6.372
MACCAb0809	*G. maculosus*	KF806076	35		CAb	Asturias	42.955	-6.372
MACCAb0810	*G. maculosus*	KF806077	35		CAb	Asturias	42.955	-6.372
MACCAb0811	*G. maculosus*	KF806078	35		CAb	Asturias	42.955	-6.372
MACCAb0812	*G. maculosus*	KF806079	35		CAb	Asturias	42.955	-6.372
MACCAb0813	*G. maculosus*	KF806080	35		CAb	Asturias	42.955	-6.372
MACCHA1708	*G. maculosus*	KF806081	36		CHA	Serra da Estrela	40.539	-7.313

Collection ID	Species	**COI** Genebank Accession Numbers	Haplotype number	Number of sequences	Location Code	Region	Latitude	Longitude
MACCHA1765	*G. maculosus*	KF806082	36		CHA	Serra da Estrela	40.539	-7.313
MACCHA0284	*G. maculosus*	KF806083	36		CHA	Serra da Estrela	40.539	-7.313
MACCHA0285	*G. maculosus*	KF806084	36		CHA	Serra da Estrela	40.539	-7.313
MACCHA0287	*G. maculosus*	KF806086	36		CHA	Serra da Estrela	40.539	-7.313
MACCHA0289	*G. maculosus*	KF806088	36	11	CHA	Serra da Estrela	40.539	-7.313
MACCHA0290	*G. maculosus*	KF806089	36		CHA	Serra da Estrela	40.539	-7.313
MACCHA0424	*G. maculosus*	KF806090	36		CHA	Serra da Estrela	40.539	-7.313
MACCHA0425	*G. maculosus*	KF806091	36		CHA	Serra da Estrela	40.539	-7.313
MACCHA0426	*G. maculosus*	KF806092	36		CHA	Serra da Estrela	40.539	-7.313
MACCHA0427	*G. maculosus*	KF806093	36		CHA	Serra da Estrela	40.539	-7.313
MACCHA0286	*G. maculosus*	KF806085	37	2	CHA	Serra da Estrela	40.539	-7.313
MACCHA0288	*G. maculosus*	KF806087	37		CHA	Serra da Estrela	40.539	-7.313
MACCAM0146	*G. maculosus*	KF806094	38		CAM	Serra da Estrela	40.383	-7.544
MACCAM1877	*G. maculosus*	KF806095	38		CAM	Serra da Estrela	40.383	-7.544
MACCAM1878	*G. maculosus*	KF806096	38		CAM	Serra da Estrela	40.383	-7.544
MACCAM1879	*G. maculosus*	KF806097	38		CAM	Serra da Estrela	40.383	-7.544
MACCAM1882	*G. maculosus*	KF806100	38		CAM	Serra da Estrela	40.383	-7.544
MACCAM0269	*G. maculosus*	KF806101	38		CAM	Serra da Estrela	40.383	-7.544
MACCAM0270	*G. maculosus*	KF806102	38		CAM	Serra da Estrela	40.383	-7.544
MACCAM0271	*G. maculosus*	KF806103	38		CAM	Serra da Estrela	40.383	-7.544
MACCAM0272	*G. maculosus*	KF806104	38	18	CAM	Serra da Estrela	40.383	-7.544
MACCAM0273	*G. maculosus*	KF806105	38		CAM	Serra da Estrela	40.383	-7.544
MACCAM0275	*G. maculosus*	KF806106	38		CAM	Serra da Estrela	40.383	-7.544
MACCAM0276	*G. maculosus*	KF806107	38		CAM	Serra da Estrela	40.383	-7.544
MACCAM0277	*G. maculosus*	KF806108	38		CAM	Serra da Estrela	40.383	-7.544
MACCAM0278	*G. maculosus*	KF806109	38		CAM	Serra da Estrela	40.383	-7.544
MACCAM0279	*G. maculosus*	KF806110	38		CAM	Serra da Estrela	40.383	-7.544
MACCAM0281	*G. maculosus*	KF806111	38		CAM	Serra da Estrela	40.383	-7.544
MACCAM0282	*G. maculosus*	KF806112	38		CAM	Serra da Estrela	40.383	-7.544
MACCAM0283	*G. maculosus*	KF806113	38		CAM	Serra da Estrela	40.383	-7.544
MACCAM1880	*G. maculosus*	KF806098	39	2	CAM	Serra da Estrela	40.383	-7.544
MACCAM1881	*G. maculosus*	KF806099	39		CAM	Serra da Estrela	40.383	-7.544
MACCOa0868	*G. maculosus*	KF806114	40		COa	Cantabria	43.307	-5.053
MACCOa0869	*G. maculosus*	KF806115	40		COa	Cantabria	43.307	-5.053
MACCOa0870	*G. maculosus*	KF806116	40	5	COa	Cantabria	43.307	-5.053
MACCOa0876	*G. maculosus*	KF806122	40		COa	Cantabria	43.307	-5.053
MACCOa0877	*G. maculosus*	KF806123	40		COa	Cantabria	43.307	-5.053
MACCOa0871	*G. maculosus*	KF806117	41		COa	Cantabria	43.307	-5.053
MACCOa0872	*G. maculosus*	KF806118	41		COa	Cantabria	43.307	-5.053
MACCOa0874	*G. maculosus*	KF806120	41	5	COa	Cantabria	43.307	-5.053
MACCOa0875	*G. maculosus*	KF806121	41		COa	Cantabria	43.307	-5.053
MACCOa0878	*G. maculosus*	KF806124	41		COa	Cantabria	43.307	-5.053
MACCOa0873	*G. maculosus*	KF806119	42	2	COa	Cantabria	43.307	-5.053
MACCOb1116	*G. maculosus*	KF806126	42		COb	Cantabria	43.307	-5.053
MACCOa0879	*G. maculosus*	KF806125	43		COa	Cantabria	43.307	-5.053
MACSAA0714	*G. maculosus*	KF806220	43		SAA	Picos de Europa	43.238	-4.227
MACSAA0715	*G. maculosus*	KF806221	43		SAA	Picos de Europa	43.238	-4.227
MACSAA0716	*G. maculosus*	KF806222	43		SAA	Picos de Europa	43.238	-4.227
MACSAA0717	*G. maculosus*	KF806223	43		SAA	Picos de Europa	43.238	-4.227
MACSAA0718	*G. maculosus*	KF806224	43		SAA	Picos de Europa	43.238	-4.227
MACSAA0719	*G. maculosus*	KF806225	43		SAA	Picos de Europa	43.238	-4.227
MACSAA0720	*G. maculosus*	KF806226	43	15	SAA	Picos de Europa	43.238	-4.227
MACSAA0721	*G. maculosus*	KF806227	43		SAA	Picos de Europa	43.238	-4.227
MACSAA0722	*G. maculosus*	KF806228	43		SAA	Picos de Europa	43.238	-4.227
MACSAA0724	*G. maculosus*	KF806230	43		SAA	Picos de Europa	43.238	-4.227
MACSAA0725	*G. maculosus*	KF806231	43		SAA	Picos de Europa	43.238	-4.227
MACSAA0726	*G. maculosus*	KF806232	43		SAA	Picos de Europa	43.238	-4.227
MACSAA0728	*G. maculosus*	KF806234	43		SAA	Picos de Europa	43.238	-4.227
MACSAA0729	*G. maculosus*	KF806235	43		SAA	Picos de Europa	43.238	-4.227
MACCOb1117	*G. maculosus*	KF806127	44	1	COb	Cantabria	43.341	-5.084
MACGUA0230	*G. maculosus*	KF806128	45		GUA	Serra da Estrela	40.535	-7.273
MACGUA0401	*G. maculosus*	KF806129	45		GUA	Serra da Estrela	40.535	-7.273
MACGUA0402	*G. maculosus*	KF806130	45		GUA	Serra da Estrela	40.535	-7.273
MACGUA0403	*G. maculosus*	KF806131	45		GUA	Serra da Estrela	40.535	-7.273
MACGUA0404	*G. maculosus*	KF806132	45		GUA	Serra da Estrela	40.535	-7.273
MACGUA0405	*G. maculosus*	KF806133	45		GUA	Serra da Estrela	40.535	-7.273
MACGUA0406	*G. maculosus*	KF806134	45		GUA	Serra da Estrela	40.535	-7.273
MACGUA0408	*G. maculosus*	KF806135	45	16	GUA	Serra da Estrela	40.535	-7.273
MACGUA0409	*G. maculosus*	KF806136	45		GUA	Serra da Estrela	40.535	-7.273
MACGUA0410	*G. maculosus*	KF806137	45		GUA	Serra da Estrela	40.535	-7.273
MACGUA0411	*G. maculosus*	KF806138	45		GUA	Serra da Estrela	40.535	-7.273
MACGUA0412	*G. maculosus*	KF806139	45		GUA	Serra da Estrela	40.535	-7.273
MACGUA0413	*G. maculosus*	KF806140	45		GUA	Serra da Estrela	40.535	-7.273
MACGUA0414	*G. maculosus*	KF806141	45		GUA	Serra da Estrela	40.535	-7.273
MACGUA0415	*G. maculosus*	KF806142	45		GUA	Serra da Estrela	40.535	-7.273
MACGUA0416	*G. maculosus*	KF806143	45		GUA	Serra da Estrela	40.535	-7.273
MACGEf1499	*G. maculosus*	KF806144	46		GEf	Geres	41.793	-8.138
MACGEf0817	*G. maculosus*	KF806145	46		GEf	Geres	41.793	-8.138
MACGEf0818	*G. maculosus*	KF806146	46	6	GEf	Geres	41.793	-8.138

Collection ID	Species	**COI** Genebank Accession Numbers	Haplotype number	Number of sequences	Location Code	Region	Latitude	Longitude
MACGEf0819	*G. maculosus*	KF806147	46	[illegible]	GEf	Geres	41.793	-8.138
MACGEf0822	*G. maculosus*	KF806150	46		GEf	Geres	41.793	-8.138
MACGEf0823	*G. maculosus*	KF806151	46		GEf	Geres	41.793	-8.138
MACGEf0820	*G. maculosus*	KF806148	47	2	GEf	Geres	41.793	-8.138
MACGEf0821	*G. maculosus*	KF806149	47		GEf	Geres	41.793	-8.138
MACWAT0428	*G. maculosus*	KF806152	48		WAT	Ireland	51.829	-10.175
MACWAT0429	*G. maculosus*	KF806153			WAT	Ireland	51.829	-10.175
MACWAT0430	*G. maculosus*	KF806154			WAT	Ireland	51.829	-10.175
MACWAT0431	*G. maculosus*	KF806155			WAT	Ireland	51.829	-10.175
MACWAT0432	*G. maculosus*	KF806156			WAT	Ireland	51.829	-10.175
MACWAT0433	*G. maculosus*	KF806157			WAT	Ireland	51.829	-10.175
MACWAT0434	*G. maculosus*	KF806158			WAT	Ireland	51.829	-10.175
MACWAT0435	*G. maculosus*	KF806159		15	WAT	Ireland	51.829	-10.175
MACWAT0436	*G. maculosus*	KF806160			WAT	Ireland	51.829	-10.175
MACWAT0437	*G. maculosus*	KF806161			WAT	Ireland	51.829	-10.175
MACWAT0438	*G. maculosus*	KF806162			WAT	Ireland	51.829	-10.175
MACWAT0439	*G. maculosus*	KF806163			WAT	Ireland	51.829	-10.175
MACWAT0440	*G. maculosus*	KF806164			WAT	Ireland	51.829	-10.175
MACWAT0441	*G. maculosus*	KF806165			WAT	Ireland	51.829	-10.175
MACWAT0442	*G. maculosus*	KF806166			WAT	Ireland	51.829	-10.175
MACMAN1231	*G. maculosus*	KF806167	49		MAN	Ourense	42.310	-7.237
MACMAN1232	*G. maculosus*	KF806168	49		MAN	Ourense	42.310	-7.237
MACMAN1233	*G. maculosus*	KF806169	49		MAN	Ourense	42.310	-7.237
MACMAN1234	*G. maculosus*	KF806170	49		MAN	Ourense	42.310	-7.237
MACMAN1235	*G. maculosus*	KF806171	49		MAN	Ourense	42.310	-7.237
MACMAN1236	*G. maculosus*	KF806172	49	11	MAN	Ourense	42.310	-7.237
MACMAN1237	*G. maculosus*	KF806173	49		MAN	Ourense	42.310	-7.237
MACMAN1238	*G. maculosus*	KF806174	49		MAN	Ourense	42.310	-7.237
MACMAN1239	*G. maculosus*	KF806175	49		MAN	Ourense	42.310	-7.237
MACMAN1241	*G. maculosus*	KF806177	49		MAN	Ourense	42.310	-7.237
MACMAN1243	*G. maculosus*	KF806179	49		MAN	Ourense	42.310	-7.237
MACMAN1240	*G. maculosus*	KF806176	50	2	MAN	Ourense	42.310	-7.237
MACMAN1242	*G. maculosus*	KF806178	50		MAN	Ourense	42.310	-7.237
MACHOb0357	*G. maculosus*	KF806180	51		HOb	Geres	41.834	-8.119
MACHOb0358	*G. maculosus*	KF806181	51		HOb	Geres	41.834	-8.119
MACHOb0359	*G. maculosus*	KF806182	51		HOb	Geres	41.834	-8.119
MACHOb0929	*G. maculosus*	KF806183	51	8	HOb	Geres	41.834	-8.119
MACHOb0930	*G. maculosus*	KF806184	51		HOb	Geres	41.834	-8.119
MACHOb0931	*G. maculosus*	KF806185	51		HOb	Geres	41.834	-8.119
MACHOb0933	*G. maculosus*	KF806187	51		HOb	Geres	41.834	-8.119
MACHOb0934	*G. maculosus*	KF806188	51		HOb	Geres	41.834	-8.119
MACHOb0932	*G. maculosus*	KF806186	52	1	HOb	Geres	41.834	-8.119
MACPOR0232	*G. maculosus*	KF806189	53	1	POR	Viana do Castelo	41.705	-8.792
MACSEE0217	*G. maculosus*	KF806190	54		SEE	Viana do Castelo	41.774	-8.619
MACSEE0218	*G. maculosus*	KF806191	54		SEE	Viana do Castelo	41.774	-8.619
MACSEE0219	*G. maculosus*	KF806192	54		SEE	Viana do Castelo	41.774	-8.619
MACSEE0221	*G. maculosus*	KF806194	54		SEE	Viana do Castelo	41.774	-8.619
MACSEE0222	*G. maculosus*	KF806195	54		SEE	Viana do Castelo	41.774	-8.619
MACSEE0223	*G. maculosus*	KF806196	54		SEE	Viana do Castelo	41.774	-8.619
MACSEE0224	*G. maculosus*	KF806198	54	13	SEE	Viana do Castelo	41.774	-8.619
MACSEE0226	*G. maculosus*	KF806214	54		SEE	Viana do Castelo	41.774	-8.619
MACSEE0234	*G. maculosus*	KF806215	54		SEE	Viana do Castelo	41.774	-8.619
MACSEE0235	*G. maculosus*	KF806216	54		SEE	Viana do Castelo	41.774	-8.619
MACSEE0236	*G. maculosus*	KF806217	54		SEE	Viana do Castelo	41.774	-8.619
MACSEE0237	*G. maculosus*	KF806218	54		SEE	Viana do Castelo	41.774	-8.619
MACSEE0238	*G. maculosus*	KF806219	54		SEE	Viana do Castelo	41.774	-8.619
MACSEE0220	*G. maculosus*	KF806193	55	2	SEE	Viana do Castelo	41.774	-8.619
MACSEE0225	*G. maculosus*	KF806209	55		SEE	Viana do Castelo	41.774	-8.619
MACSEb0239	*G. maculosus*	KF806197	56		SEb	Serra da Estrela	40.414	-7.587
MACSEb0240	*G. maculosus*	KF806199	56		SEb	Serra da Estrela	40.414	-7.587
MACSEb0241	*G. maculosus*	KF806200	56		SEb	Serra da Estrela	40.414	-7.587
MACSEb0242	*G. maculosus*	KF806201	56		SEb	Serra da Estrela	40.414	-7.587
MACSEb0243	*G. maculosus*	KF806202	56		SEb	Serra da Estrela	40.414	-7.587
MACSEb0244	*G. maculosus*	KF806203	56		SEb	Serra da Estrela	40.414	-7.587
MACSEb0245	*G. maculosus*	KF806204	56		SEb	Serra da Estrela	40.414	-7.587
MACSEb0246	*G. maculosus*	KF806205	56	15	SEb	Serra da Estrela	40.414	-7.587
MACSEb0247	*G. maculosus*	KF806206	56		SEb	Serra da Estrela	40.414	-7.587
MACSEb0248	*G. maculosus*	KF806207	56		SEb	Serra da Estrela	40.414	-7.587
MACSEb0249	*G. maculosus*	KF806208	56		SEb	Serra da Estrela	40.414	-7.587
MACSEb0250	*G. maculosus*	KF806210	56		SEb	Serra da Estrela	40.414	-7.587
MACSEb0251	*G. maculosus*	KF806211	56		SEb	Serra da Estrela	40.414	-7.587
MACSEb0252	*G. maculosus*	KF806212	56		SEb	Serra da Estrela	40.414	-7.587
MACSEb0253	*G. maculosus*	KF806213	56		SEb	Serra da Estrela	40.414	-7.587
MACSAA0723	*G. maculosus*	KF806229	57	1	SAA	Picos de Europa	43.238	-4.227
MACSAA0727	*G. maculosus*	KF806233	58	1	SAA	Picos de Europa	43.238	-4.227
MACCOM0841	*G. maculosus*	KF806236	59		COM	Coruña	42.878	-8.555
MACCOM0844	*G. maculosus*	KF806239	59		COM	Coruña	42.878	-8.555
MACCOM0845	*G. maculosus*	KF806240	59		COM	Coruña	42.878	-8.555
MACCOM0846	*G. maculosus*	KF806241	59		COM	Coruña	42.878	-8.555

Collection ID	Species	**COI** Genebank Accession Numbers	Haplotype number	Number of sequences	Location Code	Region	Latitude	Longitude
MACCOM0847	*G. maculosus*	KF806242	59		COM	Coruña	42.878	-8.555
MACCOM0848	*G. maculosus*	KF806243	59	12	COM	Coruña	42.878	-8.555
MACCOM0849	*G. maculosus*	KF806244	59		COM	Coruña	42.878	-8.555
MACCOM0850	*G. maculosus*	KF806245	59		COM	Coruña	42.878	-8.555
MACCOM0851	*G. maculosus*	KF806246	59		COM	Coruña	42.878	-8.555
MACCOM0852	*G. maculosus*	KF806247	59		COM	Coruña	42.878	-8.555
MACCOM0853	*G. maculosus*	KF806248	59		COM	Coruña	42.878	-8.555
MACCOM0855	*G. maculosus*	KF806250	59		COM	Coruña	42.878	-8.555
MACCOM0842	*G. maculosus*	KF806237	60		COM	Coruña	42.878	-8.555
MACCOM0843	*G. maculosus*	KF806238	60	4	COM	Coruña	42.878	-8.555
MACCOM0854	*G. maculosus*	KF806249	60		COM	Coruña	42.878	-8.555
MACCOM0856	*G. maculosus*	KF806251	60		COM	Coruña	42.878	-8.555
MACMAR0762	*G. maculosus*	KF806252	61		MAR	Picos de Europa	43.388	-4.110
MACMAR0763	*G. maculosus*	KF806253	61		MAR	Picos de Europa	43.388	-4.110
MACMAR0764	*G. maculosus*	KF806254	61		MAR	Picos de Europa	43.388	-4.110
MACMAR0765	*G. maculosus*	KF806255	61		MAR	Picos de Europa	43.388	-4.110
MACMAR0766	*G. maculosus*	KF806256	61		MAR	Picos de Europa	43.388	-4.110
MACMAR0767	*G. maculosus*	KF806257	61		MAR	Picos de Europa	43.388	-4.110
MACMAR0768	*G. maculosus*	KF806258	61		MAR	Picos de Europa	43.388	-4.110
MACMAR0769	*G. maculosus*	KF806259	61	15	MAR	Picos de Europa	43.388	-4.110
MACMAR0770	*G. maculosus*	KF806260	61		MAR	Picos de Europa	43.388	-4.110
MACMAR0771	*G. maculosus*	KF806261	61		MAR	Picos de Europa	43.388	-4.110
MACMAR0772	*G. maculosus*	KF806262	61		MAR	Picos de Europa	43.388	-4.110
MACMAR0773	*G. maculosus*	KF806263	61		MAR	Picos de Europa	43.388	-4.110
MACMAR0774	*G. maculosus*	KF806264	61		MAR	Picos de Europa	43.388	-4.110
MACMAR0775	*G. maculosus*	KF806265	61		MAR	Picos de Europa	43.388	-4.110
MACMAR0776	*G. maculosus*	KF806266	61		MAR	Picos de Europa	43.388	-4.110
MACVOa0967	*G. maculosus*	KF806267	62	1	VOa	Vouzela	40.721	-8.109
MACVOa0968	*G. maculosus*	KF806268	63		VOa	Vouzela	40.721	-8.109
MACVOa0969	*G. maculosus*	KF806269	63	3	VOa	Vouzela	40.721	-8.109
MACVOa0972	*G. maculosus*	KF806272	63		VOa	Vouzela	40.721	-8.109
MACVOa0970	*G. maculosus*	KF806270	64		VOa	Vouzela	40.721	-8.109
MACVOa0971	*G. maculosus*	KF806271	64	3	VOa	Vouzela	40.721	-8.109
MACVOa0973	*G. maculosus*	KF806273	64		VOa	Vouzela	40.721	-8.109
MALBRE0184	*G. malagensis*	KF806274	65		BRE	Sesimbra	38.541	-9.030
MALBRE0183	*G. malagensis*	KF806275	65		BRE	Sesimbra	38.541	-9.030
MALBRE1224	*G. malagensis*	KF806276	65		BRE	Sesimbra	38.541	-9.030
MALBRE1225	*G. malagensis*	KF806277	65		BRE	Sesimbra	38.541	-9.030
MALBRE1226	*G. malagensis*	KF806278	65		BRE	Sesimbra	38.541	-9.030
MALBRE1229	*G. malagensis*	KF806279	65		BRE	Sesimbra	38.541	-9.030
MALBRE0181	*G. malagensis*	KF806280	65		BRE	Sesimbra	38.541	-9.030
MALBRE0182	*G. malagensis*	KF806281	65		BRE	Sesimbra	38.541	-9.030
MALSSb1831	*G. malagensis*	KF806282	65		SSb	Sesimbra	38.479	-9.113
MALSSb1832	*G. malagensis*	KF806283	65		SSb	Sesimbra	38.479	-9.113
MALSSb1833	*G. malagensis*	KF806284	65		SSb	Sesimbra	38.479	-9.113
MALSSb1834	*G. malagensis*	KF806285	65		SSb	Sesimbra	38.479	-9.113
MALSSb1835	*G. malagensis*	KF806286	65		SSb	Sesimbra	38.479	-9.113
MALSSb1836	*G. malagensis*	KF806287	65		SSb	Sesimbra	38.479	-9.113
MALSSb1837	*G. malagensis*	KF806288	65		SSb	Sesimbra	38.479	-9.113
MALSSb1838	*G. malagensis*	KF806289	65		SSb	Sesimbra	38.479	-9.113
MALSSb1839	*G. malagensis*	KF806290	65		SSb	Sesimbra	38.479	-9.113
MALSSb1840	*G. malagensis*	KF806291	65		SSb	Sesimbra	38.479	-9.113
MALSSb1841	*G. malagensis*	KF806292	65	37	SSb	Sesimbra	38.479	-9.113
MALSSb1842	*G. malagensis*	KF806293	65		SSb	Sesimbra	38.479	-9.113
MALSSb1843	*G. malagensis*	KF806294	65		SSb	Sesimbra	38.479	-9.113
MALSSb1844	*G. malagensis*	KF806295	65		SSb	Sesimbra	38.479	-9.113
MALCLb1535	*G. malagensis*	KF806302	65		CLb	Huelva	37.679	-6.650
MALCLb1536	*G. malagensis*	KF806303	65		CLb	Huelva	37.679	-6.650
MALCLb1542	*G. malagensis*	KF806309	65		CLb	Huelva	37.679	-6.650
MALCLb1543	*G. malagensis*	KF806310	65		CLb	Huelva	37.679	-6.650
MALSSa0154	*G. malagensis*	KF806350	65		SSa	Sesimbra	38.486	-9.121
MALSSa0155	*G. malagensis*	KF806351	65		SSa	Sesimbra	38.486	-9.121
MALSSa0156	*G. malagensis*	KF806352	65		SSa	Sesimbra	38.486	-9.121
MALSSa0159	*G. malagensis*	KF806353	65		SSa	Sesimbra	38.486	-9.121
MALSSa0160	*G. malagensis*	KF806354	65		SSa	Sesimbra	38.486	-9.121
MALSSa0161	*G. malagensis*	KF806355	65		SSa	Sesimbra	38.486	-9.121
MALSSa0681	*G. malagensis*	KF806356	65		SSa	Sesimbra	38.486	-9.121
MALSSa0682	*G. malagensis*	KF806357	65		SSa	Sesimbra	38.486	-9.121
MALSSa0683	*G. malagensis*	KF806358	65		SSa	Sesimbra	38.486	-9.121
MALSSa0498	*G. malagensis*	KF806362	65		SSa	Sesimbra	38.486	-9.121
MALSSa0153	*G. malagensis*	KF806363	65		SSa	Sesimbra	38.486	-9.121
MALCLa0390	*G. malagensis*	KF806296	66		CLa	Huelva	37.679	-6.653
MALCLb1530	*G. malagensis*	KF806297	66		CLb	Huelva	37.679	-6.650
MALCLb1537	*G. malagensis*	KF806304	66		CLb	Huelva	37.679	-6.650
MALCLb1538	*G. malagensis*	KF806305	66		CLb	Huelva	37.679	-6.650
MALCLb1539	*G. malagensis*	KF806306	66		CLb	Huelva	37.679	-6.650
MALGIa0510	*G. malagensis*	KF806333	66		GIa	Gibraltar	36.131	-5.351
MALGIa0512	*G. malagensis*	KF806335	66	13	GIa	Gibraltar	36.131	-5.351
MALGIa0517	*G. malagensis*	KF806340	66		GIa	Gibraltar	36.131	-5.351

Collection ID	Species	**COI** Genebank Accession Numbers	Haplotype number	Number of sequences	Location Code	Region	Latitude	Longitude
MALGIa0518	*G. malagensis*	KF806341	66		GIa	Gibraltar	36.131	-5.351
MALGIa0520	*G. malagensis*	KF806343	66		GIa	Gibraltar	36.131	-5.351
MALGIa0523	*G. malagensis*	KF806345	66		GIa	Gibraltar	36.131	-5.351
MALGIa0601	*G. malagensis*	KF806347	66		GIa	Gibraltar	36.131	-5.351
MALGIa1603	*G. malagensis*	KF806349	66		GIa	Gibraltar	36.131	-5.351
MALCLb1531	*G. malagensis*	KF806298	67	1	CLb	Gibraltar	37.679	-6.650
MALCLb1532	*G. malagensis*	KF806299	68		CLb	Gibraltar	37.679	-6.650
MALCLb1533	*G. malagensis*	KF806300	68		CLb	Gibraltar	37.679	-6.650
MALCLb1534	*G. malagensis*	KF806301	68	5	CLb	Gibraltar	37.679	-6.650
MALCLb1540	*G. malagensis*	KF806307	68		CLb	Gibraltar	37.679	-6.650
MALCLb1541	*G. malagensis*	KF806308	68		CLb	Gibraltar	37.679	-6.650
MALJER0563	*G. malagensis*	KF806311	69		JER	Gibraltar	36.255	-5.582
MALJER0564	*G. malagensis*	KF806312	69	4	JER	Gibraltar	36.255	-5.582
MALJER0566	*G. malagensis*	KF806314	69		JER	Gibraltar	36.255	-5.582
MALJER0568	*G. malagensis*	KF806316	69		JER	Gibraltar	36.255	-5.582
MALJER0565	*G. malagensis*	KF806313	70	1	JER	Gibraltar	36.255	-5.582
MALJER0567	*G. malagensis*	KF806315	71	1	JER	Gibraltar	36.255	-5.582
MALGUE1845	*G. malagensis*	KF806317	72		GUE	Faro	37.098	-7.922
MALGUE1846	*G. malagensis*	KF806318	72		GUE	Faro	37.098	-7.922
MALGUE1861	*G. malagensis*	KF806319	72		GUE	Faro	37.098	-7.922
MALGUE1862	*G. malagensis*	KF806320	72		GUE	Faro	37.098	-7.922
MALGUE1876	*G. malagensis*	KF806321	72		GUE	Faro	37.098	-7.922
MALGUE1883	*G. malagensis*	KF806322	72		GUE	Faro	37.098	-7.922
MALGUE1884	*G. malagensis*	KF806323	72		GUE	Faro	37.098	-7.922
MALGUE1885	*G. malagensis*	KF806324	72		GUE	Faro	37.098	-7.922
MALGUE1798	*G. malagensis*	KF806325	72		GUE	Faro	37.098	-7.922
MALGUE1803	*G. malagensis*	KF806326	72		GUE	Faro	37.098	-7.922
MALGUE1804	*G. malagensis*	KF806327	72		GUE	Faro	37.098	-7.922
MALGUE1805	*G. malagensis*	KF806328	72		GUE	Faro	37.098	-7.922
MALGUE1806	*G. malagensis*	KF806329	72	25	GUE	Faro	37.098	-7.922
MALGUE1807	*G. malagensis*	KF806330	72		GUE	Faro	37.098	-7.922
MALSSa0684	*G. malagensis*	KF806359	72		SSa	Sesimbra	38.486	-9.121
MALSSa0497	*G. malagensis*	KF806361	72		SSa	Sesimbra	38.486	-9.121
MALARR0977	*G. malagensis*	KF806364	72		ARR	Aljezur	37.296	-8.865
MALARR0978	*G. malagensis*	KF806365	72		ARR	Aljezur	37.296	-8.865
MALARR0979	*G. malagensis*	KF806366	72		ARR	Aljezur	37.296	-8.865
MALARR0980	*G. malagensis*	KF806367	72		ARR	Aljezur	37.296	-8.865
MALARR0981	*G. malagensis*	KF806368	72		ARR	Aljezur	37.296	-8.865
MALARR0982	*G. malagensis*	KF806369	72		ARR	Aljezur	37.296	-8.865
MALARR0983	*G. malagensis*	KF806370	72		ARR	Aljezur	37.296	-8.865
MALARR0984	*G. malagensis*	KF806371	72		ARR	Aljezur	37.296	-8.865
MALARR0985	*G. malagensis*	KF806372	72		ARR	Aljezur	37.296	-8.865
MALGIb0605	*G. malagensis*	KF806331	73		GIb	Gibraltar	36.118	-5.345
MALGIa0511	*G. malagensis*	KF806334	73		GIa	Gibraltar	36.131	-5.351
MALGIa0514	*G. malagensis*	KF806337	73	5	GIa	Gibraltar	36.131	-5.351
MALGIa0515	*G. malagensis*	KF806338	73		GIa	Gibraltar	36.131	-5.351
MALGIa0524	*G. malagensis*	KF806346	73		GIa	Gibraltar	36.131	-5.351
MALGIb0606	*G. malagensis*	KF806332	74	1	GIb	Gibraltar	36.118	-5.345
MALGIa0513	*G. malagensis*	KF806336	75	1	GIa	Gibraltar	36.131	-5.351
MALGIa0516	*G. malagensis*	KF806339	76	2	GIa	Gibraltar	36.131	-5.351
MALGIa0519	*G. malagensis*	KF806342	76		GIa	Gibraltar	36.131	-5.351
MALGIa0522	*G. malagensis*	KF806344	77	2	GIa	Gibraltar	36.131	-5.351
MALGIa0602	*G. malagensis*	KF806348	77		GIa	Gibraltar	36.131	-5.351
MALSSa0231	*G. malagensis*	KF806360	78	1	SSa	Sesimbra	38.486	-9.121
OLITOa1151	*G.oliveirae*	KF806373	79		TOa	Montes de Toledo	39.563	-4.585
OLITOa1177	*G.oliveirae*	KF806376	79	4	TOa	Montes de Toledo	39.563	-4.585
OLITOa1178	*G.oliveirae*	KF806377	79		TOa	Montes de Toledo	39.563	-4.585
OLITOa1179	*G.oliveirae*	KF806378	79		TOa	Montes de Toledo	39.563	-4.585
OLITOa1164	*G.oliveirae*	KF806374	80	1	TOa	Montes de Toledo	39.563	-4.585
OLITOa1165	*G.oliveirae*	KF806375	81		TOa	Montes de Toledo	39.563	-4.585
OLITOa1475	*G.oliveirae*	KF806383	81	3	TOa	Montes de Toledo	39.563	-4.585
OLITOa0421	*G.oliveirae*	KF806385	81		TOa	Montes de Toledo	39.563	-4.585
OLITOa1180	*G.oliveirae*	KF806379	82	1	TOa	Montes de Toledo	39.563	-4.585
OLITOa1181	*G.oliveirae*	KF806380	83	1	TOa	Montes de Toledo	39.563	-4.585
OLITOa1182	*G.oliveirae*	KF806381	84	2	TOa	Montes de Toledo	39.563	-4.585
OLITOa1808	*G.oliveirae*	KF806384	84		TOa	Montes de Toledo	39.563	-4.585
OLITOa1280	*G.oliveirae*	KF806382	85	1	TOa	Montes de Toledo	39.563	-4.585
OLITOb1118	*G.oliveirae*	KF806386	86		TOb	Montes de Toledo	39.585	-4.527
OLITOb1119	*G.oliveirae*	KF806387	86	3	TOb	Montes de Toledo	39.585	-4.527
OLITOb1121	*G.oliveirae*	KF806389	86		TOb	Montes de Toledo	39.585	-4.527
OLITOb1120	*G.oliveirae*	KF806388	87		TOb	Montes de Toledo	39.585	-4.527
OLITOb1124	*G.oliveirae*	KF806392	87		TOb	Montes de Toledo	39.585	-4.527
OLITOb1126	*G.oliveirae*	KF806394	87	5	TOb	Montes de Toledo	39.585	-4.527
OLITOb1128	*G.oliveirae*	KF806396	87		TOb	Montes de Toledo	39.585	-4.527
OLITOb1129	*G.oliveirae*	KF806397	87		TOb	Montes de Toledo	39.585	-4.527
OLITOb1122	*G.oliveirae*	KF806390	88	1	TOb	Montes de Toledo	39.585	-4.527
OLITOb1123	*G.oliveirae*	KF806391	89	1	TOb	Montes de Toledo	39.585	-4.527
OLITOb1125	*G.oliveirae*	KF806393	90	1	TOb	Montes de Toledo	39.585	-4.527
OLITOb1127	*G.oliveirae*	KF806395	91	1	TOb	Montes de Toledo	39.585	-4.527

Collection ID	Species	**COI** Genebank Accession Numbers	Haplotype number	Number of sequences	Location Code	Region	Latitude	Longitude
OLITOb1130	*G.oliveirae*	KF806398	92	1	TOb	Montes de Toledo	39.585	-4.527
OLITOb1131	*G.oliveirae*	KF806399	93	1	TOb	Montes de Toledo	39.585	-4.527
OLITOb0488	*G.oliveirae*	KF806400	94	1	TOb	Montes de Toledo	39.585	-4.527
OLIGRa1762	*G.oliveirae*	KF806401	95	10	GRa	Sierra de Gredos	40.322	-5.014
OLIGRa1763	*G.oliveirae*	KF806402	95		GRa	Sierra de Gredos	40.322	-5.014
OLIGRa1802	*G.oliveirae*	KF806403	95		GRa	Sierra de Gredos	40.322	-5.014
OLIGRb1752	*G.oliveirae*	KF806406	95		GRb	Sierra de Gredos	40.308	-5.000
OLIGRb1755	*G.oliveirae*	KF806409	95		GRb	Sierra de Gredos	40.308	-5.000
OLIGRb1758	*G.oliveirae*	KF806412	95		GRb	Sierra de Gredos	40.308	-5.000
OLIGRb1759	*G.oliveirae*	KF806413	95		GRb	Sierra de Gredos	40.308	-5.000
OLIGRb1760	*G.oliveirae*	KF806414	95		GRb	Sierra de Gredos	40.308	-5.000
OLIGRb1761	*G.oliveirae*	KF806415	95		GRb	Sierra de Gredos	40.308	-5.000
OLIGRb1764	*G.oliveirae*	KF806416	95		GRb	Sierra de Gredos	40.308	-5.000
OLIGRb1145	*G.oliveirae*	KF806404	96	2	GRb	Sierra de Gredos	40.308	-5.000
OLIGRb1757	*G.oliveirae*	KF806411	96		GRb	Sierra de Gredos	40.308	-5.000
OLIGRb1146	*G.oliveirae*	KF806405	97	1	GRb	Sierra de Gredos	40.308	-5.000
OLIGRb1753	*G.oliveirae*	KF806407	98	1	GRb	Sierra de Gredos	40.308	-5.000
OLIGRb1754	*G.oliveirae*	KF806408	99	2	GRb	Sierra de Gredos	40.308	-5.000
OLIGRb1756	*G.oliveirae*	KF806410	99		GRb	Sierra de Gredos	40.308	-5.000
OLIGRc1775	*G.oliveirae*	KF806417	100	1	GRc	Sierra de Gredos	40.308	-5.000
OLILPa0420	*G.oliveirae*	KF806418	101	2	LPa	Sierra de Guadalupe	39.440	-5.315
OLILPa0490	*G.oliveirae*	KF806419	101		LPa	Sierra de Guadalupe	39.440	-5.315
OLILPb1778	*G.oliveirae*	KF806420	102	1	LPb	Sierra de Guadalupe	39.474	-5.368
OLILPb1779	*G.oliveirae*	KF806421	103	10	LPb	Sierra de Guadalupe	39.474	-5.368
OLILPb1781	*G.oliveirae*	KF806423	103		LPb	Sierra de Guadalupe	39.474	-5.368
OLILPb1795	*G.oliveirae*	KF806424	103		LPb	Sierra de Guadalupe	39.474	-5.368
OLILPb0370	*G.oliveirae*	KF806425	103		LPb	Sierra de Guadalupe	39.474	-5.368
OLILPb0371	*G.oliveirae*	KF806426	103		LPb	Sierra de Guadalupe	39.474	-5.368
OLILPb0378	*G.oliveirae*	KF806430	103		LPb	Sierra de Guadalupe	39.474	-5.368
OLILPb0379	*G.oliveirae*	KF806431	103		LPb	Sierra de Guadalupe	39.474	-5.368
OLILPb0380	*G.oliveirae*	KF806432	103		LPb	Sierra de Guadalupe	39.474	-5.368
OLILPb0400	*G.oliveirae*	KF806433	103		LPb	Sierra de Guadalupe	39.474	-5.368
OLILPb0489	*G.oliveirae*	KF806434	103		LPb	Sierra de Guadalupe	39.474	-5.368
OLILPb1780	*G.oliveirae*	KF806422	104	1	LPb	Sierra de Guadalupe	39.474	-5.368
OLILPb0372	*G.oliveirae*	KF806427	105	1	LPb	Sierra de Guadalupe	39.474	-5.368
OLILPb0373	*G.oliveirae*	KF806428	106	1	LPb	Sierra de Guadalupe	39.474	-5.368
OLILPb0375	*G.oliveirae*	KF806429	107	1	LPb	Sierra de Guadalupe	39.474	-5.368
OLIPEa1766	*G.oliveirae*	KF806435	108	3	PEa	Sierra de Peña de Francia	40.514	-6.157
OLIPEb1771	*G.oliveirae*	KF806436	108		PEb	Sierra de Peña de Francia	40.514	-6.157
OLIPEa0382	*G.oliveirae*	KF806437	108		PEa	Sierra de Peña de Francia	40.514	-6.157
OLISEa0422	*G.oliveirae*	KF806438	109	1	SEa	Serra da Estrela	40.461	-7.587
OLISEb1279	*G.oliveirae*	KF806439	110	1	SEb	Serra da Estrela	40.414	-7.587

Supplementary Table S4. List of species and GenBank accession numbers of available COI sequences from all Iberian endemic and non-endemic *Arion* species, other Arionidae genera, and from the outgroup used in the analyses.

Endemic Species	GenBank Accession Number
Arion nobrei	JQ041667
Arion gilvus	KF305200
Arion urbiae	AY987919
Arion baeticus	AY987871
Arion paularensis	AY987899
Arion wiktori	AY987921
Arion molinae	AY987896
Arion ponsi	KF305213
Arion lizarrustii	AY987893
Arion iratii	AY987892
Arion flagellus	AY987882
Arion fuligineus	-
Arion fagophilus	-
Arion anthracius	-
Arion magnus	-
Arion hispanicus	-
Arion luisae	-
**Non-Endemic Species**	**GenBank Accession Number**
Arion alpinus	AY987867
Arion ater	KX834786
Arion circumscriptus	AY987873
Arion distinctus	AY987874
Arion fasciatus	AY987877
Arion fuscus	AY987885
Arion hortensis	AY987888
Arion intermedius	AY987891
Arion lusitanicus	EF520642
Arion occultus	FJ348255
Arion owenii	AY987897
Arion rufus	AY987901
Arion silvaticus	AY987917
Arion subfuscus	AY987904
Arion vulgaris	KX834811
**Other Arionidae species**	**GenBank Accession Number**
Kootenaia burkei	AY382636
Hemphillia glandulosa	AY382630
Hemphillia dromedarius	AY382631
Prophysaon andersoni	KM612059
Hemphillia camelus	AY382632
**Outgroup**	**GenBank Accession Number**
Lehmannia valentiana	JX117876
Limax flavus	FJ606457

Supplementary Table S5. Collection ID, species name, accession number, sequence number, number of sequences (18S rRNA), location code, region, latitude and longitude for *Geomalacus* specimens.

Collection ID	Species	**18S** Genebank Accession Numbers	Sequence number	Number of sequences	Location Code	Region	Latitude	Longitude
OLIGRb1145	*G.oliveirae*	KF953734	1		GRb	Sierra de Gredos	40.308	-5.000
OLIGRb1146	*G.oliveirae*	KF953735	1		GRb	Sierra de Gredos	40.308	-5.000
OLIGRb1752	*G.oliveirae*	KF953736	1		GRb	Sierra de Gredos	40.308	-5.000
OLIGRb1753	*G.oliveirae*	KF953737	1		GRb	Sierra de Gredos	40.308	-5.000
OLIGRb1754	*G.oliveirae*	KF953738	1		GRb	Sierra de Gredos	40.308	-5.000
OLIGRb1756	*G.oliveirae*	KF953739	1		GRb	Sierra de Gredos	40.308	-5.000
OLIGRc1775	*G.oliveirae*	KF953740	1		GRc	Sierra de Gredos	40.308	-5.000
OLILPa0420	*G.oliveirae*	KF953741	1		LPa	Sierra de Guadalupe	39.440	-5.315
OLILPb0371	*G.oliveirae*	KF953744	1		LPb	Sierra de Guadalupe	39.474	-5.368
OLILPb0372	*G.oliveirae*	KF953745	1		LPb	Sierra de Guadalupe	39.474	-5.368
OLILPb0373	*G.oliveirae*	KF953746	1		LPb	Sierra de Guadalupe	39.474	-5.368
OLILPb0375	*G.oliveirae*	KF953747	1		LPb	Sierra de Guadalupe	39.474	-5.368
OLILPb1779	*G.oliveirae*	KF953742	1	25	LPb	Sierra de Guadalupe	39.474	-5.368
OLILPb1780	*G.oliveirae*	KF953743	1		LPb	Sierra de Guadalupe	39.474	-5.368
OLIPEa1766	*G.oliveirae*	KF953748	1		PEa	Sierra de Peña de Francia	40.514	-6.157
OLISEa0422	*G.oliveirae*	KF953749	1		SEa	Serra da Estrela	40.461	-7.587
OLITOb0488	*G.oliveirae*	KF953733	1		TOb	Montes de Toledo	39.585	-4.527
OLITOb1119	*G.oliveirae*	KF953725	1		TOb	Montes de Toledo	39.585	-4.527
OLITOb1120	*G.oliveirae*	KF953726	1		TOb	Montes de Toledo	39.585	-4.527
OLITOb1122	*G.oliveirae*	KF953727	1		TOb	Montes de Toledo	39.585	-4.527
OLITOb1123	*G.oliveirae*	KF953728	1		TOb	Montes de Toledo	39.585	-4.527
OLITOb1125	*G.oliveirae*	KF953729	1		TOb	Montes de Toledo	39.585	-4.527
OLITOb1127	*G.oliveirae*	KF953730	1		TOb	Montes de Toledo	39.585	-4.527
OLITOb1130	*G.oliveirae*	KF953731	1		TOb	Montes de Toledo	39.585	-4.527
OLITOb1131	*G.oliveirae*	KF953732	1		TOb	Montes de Toledo	39.585	-4.527
ANGMOa0147	*G. anguiformis*	KF953750	2		MOa	Monchique	37.307	-8.570
ANGMOb0261	*G. anguiformis*	KF953751	2		MOb	Monchique	37.305	-8.588
ANGMOd0340	*G. anguiformis*	KF953752	2		MOd	Monchique	37.342	-8.488
ANGMOe0505	*G. anguiformis*	KF953756	2		MOe	Monchique	37.313	-8.549
ANGMOe0529	*G. anguiformis*	KF953754	2		MOe	Monchique	37.313	-8.549
ANGMOe0532	*G. anguiformis*	KF953753	2		MOe	Monchique	37.313	-8.549
ANGMOe1149	*G. anguiformis*	KF953755	2		MOe	Monchique	37.313	-8.549
ANGMOf0548	*G. anguiformis*	KF953759	2		MOf	Monchique	37.306	-8.499
ANGMOf0549	*G. anguiformis*	KF953761	2	17	MOf	Monchique	37.306	-8.499
ANGMOf0552	*G. anguiformis*	KF953758	2		MOf	Monchique	37.306	-8.499
ANGMOf0554	*G. anguiformis*	KF953760	2		MOf	Monchique	37.306	-8.499
ANGMOf0560	*G. anguiformis*	KF953757	2		MOf	Monchique	37.306	-8.499
ANGSBb1154	*G. anguiformis*	KF953762	2		SBb	Faro	37.273	-7.875
ANGSBb1172	*G. anguiformis*	KF953763	2		SBb	Faro	37.273	-7.875
ARAARA0525	*G. anguiformis*	KF953766	2		ARA	Aracena	37.878	-6.817
ARAARA0535	*G. anguiformis*	KF953764	2		ARA	Aracena	37.878	-6.817
ARAARA0547	*G. anguiformis*	KF953765	2		ARA	Aracena	37.878	-6.817
MACBRA0492	*G. maculosus*	KF953767	3		BRA	Bragança	41.688	-6.751
MACCAa0922	*G. maculosus*	KF953768	3		CAa	Asturias	42.949	-6.395
MACCAb0806	*G. maculosus*	KF953769	3		CAb	Asturias	42.955	-6.372
MACCAM0146	*G. maculosus*	KF953770	3		CAM	Serra da Estrela	40.383	-7.544
MACCAM1880	*G. maculosus*	KF953771	3		CAM	Serra da Estrela	40.383	-7.544
MACCOa0868	*G. maculosus*	KF953772	3		COa	Cantabria	43.307	-5.053
MACCOb1116	*G. maculosus*	KF953773	3		COb	Cantabria	43.307	-5.053
MACCOM0841	*G. maculosus*	KF953786	3		COM	Coruña	42.878	-8.555
MACCOM0842	*G. maculosus*	KF953787	3		COM	Coruña	42.878	-8.555
MACGEf0820	*G. maculosus*	KF953775	3		GEf	Geres	41.793	-8.138
MACGUA0230	*G. maculosus*	KF953774	3		GUA	Serra da Estrela	40.535	-7.273
MACHOb0357	*G. maculosus*	KF953778	3		HOb	Geres	41.834	-8.119
MACHOb0932	*G. maculosus*	KF953779	3	25	HOb	Geres	41.834	-8.119
MACMAN1231	*G. maculosus*	KF953776	3		MAN	Ourense	42.310	-7.237
MACMAN1240	*G. maculosus*	KF953777	3		MAN	Ourense	42.310	-7.237
MACMAR0762	*G. maculosus*	KF953788	3		MAR	Picos de Europa	43.388	-4.110
MACPOR0232	*G. maculosus*	KF953780	3		POR	Viana do Castelo	41.705	-8.792
MACSAA0723	*G. maculosus*	KF953784	3		SAA	Picos de Europa	43.238	-4.227
MACSAA0727	*G. maculosus*	KF953785	3		SAA	Picos de Europa	43.238	-4.227
MACSEb0239	*G. maculosus*	KF953782	3		SEb	Serra da Estrela	40.414	-7.587
MACSEb0243	*G. maculosus*	KF953783	3		SEb	Serra da Estrela	40.414	-7.587
MACSEE0217	*G. maculosus*	KF953781	3		SEE	Viana do Castelo	41.774	-8.619
MACVOa0967	*G. maculosus*	KF953789	3		VOa	Vouzela	40.721	-8.109
MACVOa0968	*G. maculosus*	KF953790	3		VOa	Vouzela	40.721	-8.109
MACVOa0970	*G. maculosus*	KF953791	3		VOa	Vouzela	40.721	-8.109
MALCLb1532	*G. malagensis*	KF953799	4		CLb	Gibraltar	37.679	-6.650
MALGIa0510	*G. malagensis*	KF953803	4		GIa	Gibraltar	36.131	-5.351
MALGIa0511	*G. malagensis*	KF953804	4		GIa	Gibraltar	36.131	-5.351
MALGIa0516	*G. malagensis*	KF953805	4		GIa	Gibraltar	36.131	-5.351
MALGIa0517	*G. malagensis*	KF953806	4		GIa	Gibraltar	36.131	-5.351
MALGIa0522	*G. malagensis*	KF953807	4	17	GIa	Gibraltar	36.131	-5.351
MALGUE1798	*G. malagensis*	KF953802	4		GUE	Faro	37.098	-7.922
MALJER0563	*G. malagensis*	KF953800	4		JER	Gibraltar	36.255	-5.582
MALJER0567	*G. malagensis*	KF953801	4		JER	Gibraltar	36.255	-5.582
MALSSa0159	*G. malagensis*	KF953808	4		SSa	Sesimbra	38.486	-9.121
MALSSb1843	*G. malagensis*	KF953798	4		SSb	Sesimbra	38.479	-9.113

Box1. Workflow of the methods used to test the different diversity-dependence models to explain the observed differences in clade richness between two genera of Iberian endemic terrestrial slugs.

Hypotheses		Method	Models	Result	Conclusion
H1	Differences in clade age	BEAST dated phylogeny	Birth-death incomplete sampling<br>Tree prior	No differences in clade age (Figure 2)	No
H2	Differences in constant diversification rates	make.bd.split [diversitree]<br>[incomplete sampling taxon taken into account]		Best-fit = model 3 (Table 2).<br>Extinction rates equal to zero in both genera.<br>Equal speciation rates for the two genera.	No
H3	Combination of H1 and H2<br>Differences in clade age and diversification rates			No differences in clade age or in diversification rates	No

How to explain this contradictory result suggesting that neither differences in clade age nor unequal diversification rates explain the existence of a clade-poor and a clade-rich genera? Testing differences on how diversification rates vary over time within each clade.

Hypotheses		Method	Models	Result	Conclusion
H4	Differences in variable diversification rates within each clade	fitdAICrc [laser]	1) λA, 2) λG; 3) λA, μA; 4) λG, μG;<br>**5) *r*0A, *K*A**; 6) ***r*0G, *K*G**;<br>7) *r*0A, *xp*A; 8) *r*0G, *xp*G;<br>9) *r*0A, *r*1A; 10) *r*0G, *r*1G.	Rate variable models fit the data better than constant rate models in all three sets of models evaluated. (Table 3, 4 and 5).	
		make.bd.t [diversitree][IST]	1) λA, 2) λA, μA; 3) ***r*0A, *K*A**.		
		dd_ML [DDD] [IST]<br>[IST = incomplete sampling taxon taken into account]	1) *r*0A, *r*1A; 2) *r*0A, *r*1A, *k*A.		

If variable models are a better fit to the data genus are taken separately, a more refined diversity-dependent diversification model with decoupling of a subclade's diversification dynamics from the main clade's dynamics can indicate which parameter (λ and/or *K*) is responsible for the species number difference.

Hypotheses		Method	Models	Result	Conclusion
H5.1	λ differs between genera (models: 1,2,4,5)<br>(Higher initial speciation rate in *Arion*)	dd_KI_ML[DDD]<br>[incomplete sampling taxon taken into account]	1) *r*0A, *r*0G, μA, μG, kA, kG<br>2) *r*0A, *r*0G, μA ≈ 0, μG ≈ 0, kA, kG<br>**3) *r*0A ≈ *r*0G, μA ≈ 0, μG ≈ 0, kA, kG**<br>4) *r*0A, *r*0G, μA ≈ 0, μG ≈ 0, kA ≈ kλG<br>5) *r*0A, *r*0G, μA ≈ 0, μG ≈ 0, kA ≈ 0, kG ≈ 0<br>6) *r*0A ≈ *r*0G, μA ≈ 0, μG ≈ 0, kA ≈ 0, kG ≈ 0	Best-fit = model 3 (Table 6).<br>Equal *r*0 between genus	No
H5.2	K differs between genera (models: 1,2,3)<br>(Weaker decline of speciation rate in species-rich clade?)			Different k for each genus	Yes
H6	Combination of H5.1 and H5.2				No

A, Arion Clade 1; G, Geomalacus clade; λ, speciation rate; μ, extinction rate when applicable; *K*, carrying capacity parameter of DDL and DDD models; *r*0 (= λ0-μ0), initial net diversification rate for diversity dependent linear (DDL), diversity dependent exponential (DDX), and yule2rate (or constant net diversification rate for pure-birth and birth-death model) models; *r*1 = net diversification rate after the first shift at time *t*1; *xp*, exponent of DDX model; *k*, parameter of the exponential change in speciation rate for the models SPVAR.

Supplementary Figure S1.
Phylogenetic relationships of Geomalacus based on a Bayesian analysis of a combined data set (74 taxa; COI: 594 bp and 18S rRNA: 524 bp). Numbers at the nodes are from top to bottom: Bayesian posterior probabilities (BPP) and bootstrap proportions (BP), respectively. Only values above 50% (BP) and 70% (BPP) are represented.

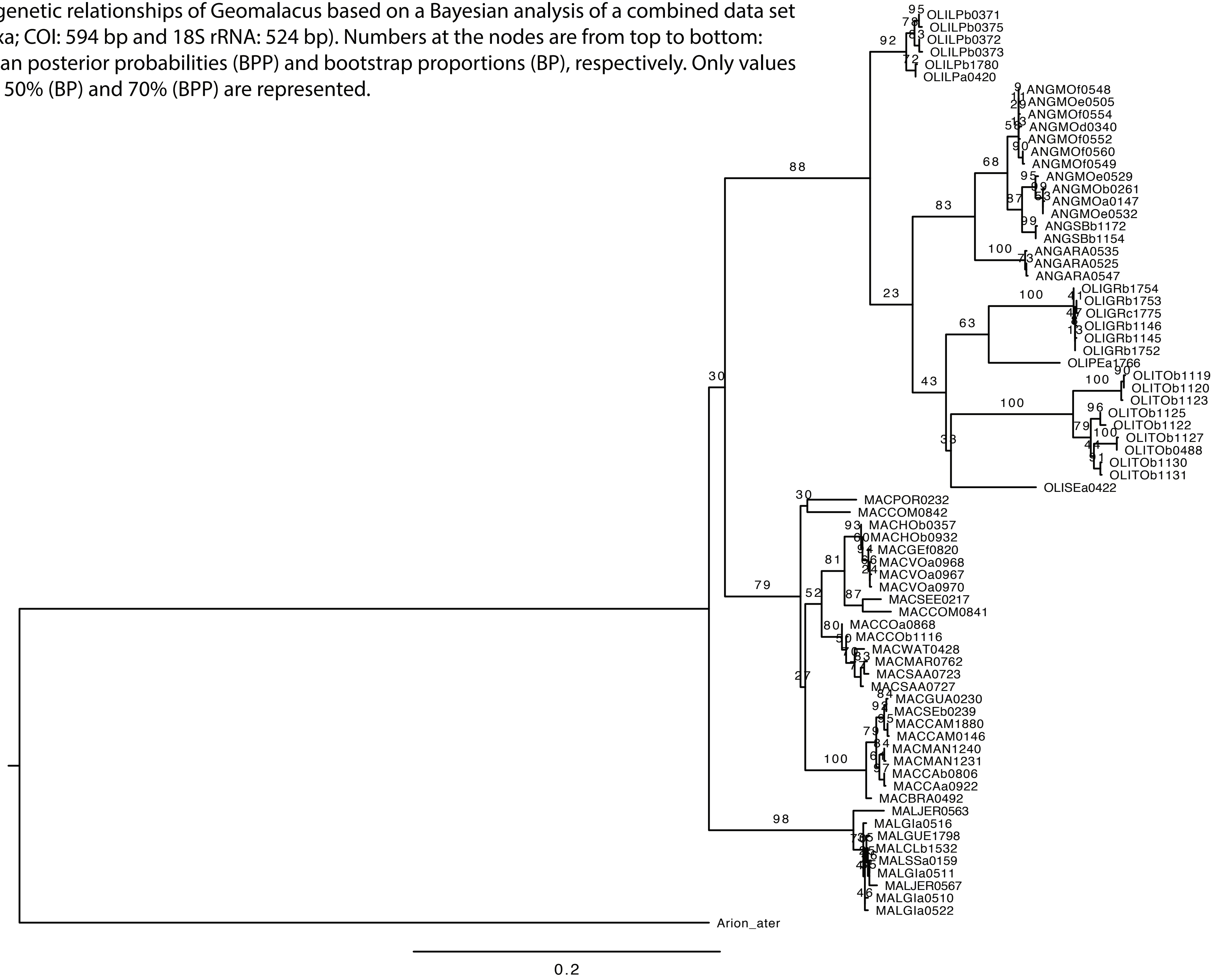

Supplementary Figure S2. Phylogenetic relationships of Arionidae based on a Bayesian analysis of cytochrome oxidase subunit I (42 taxa: 523 bp). Numbers at the nodes are Bayesian posterior probabilities. Only values above 70% (BPP) are represented.

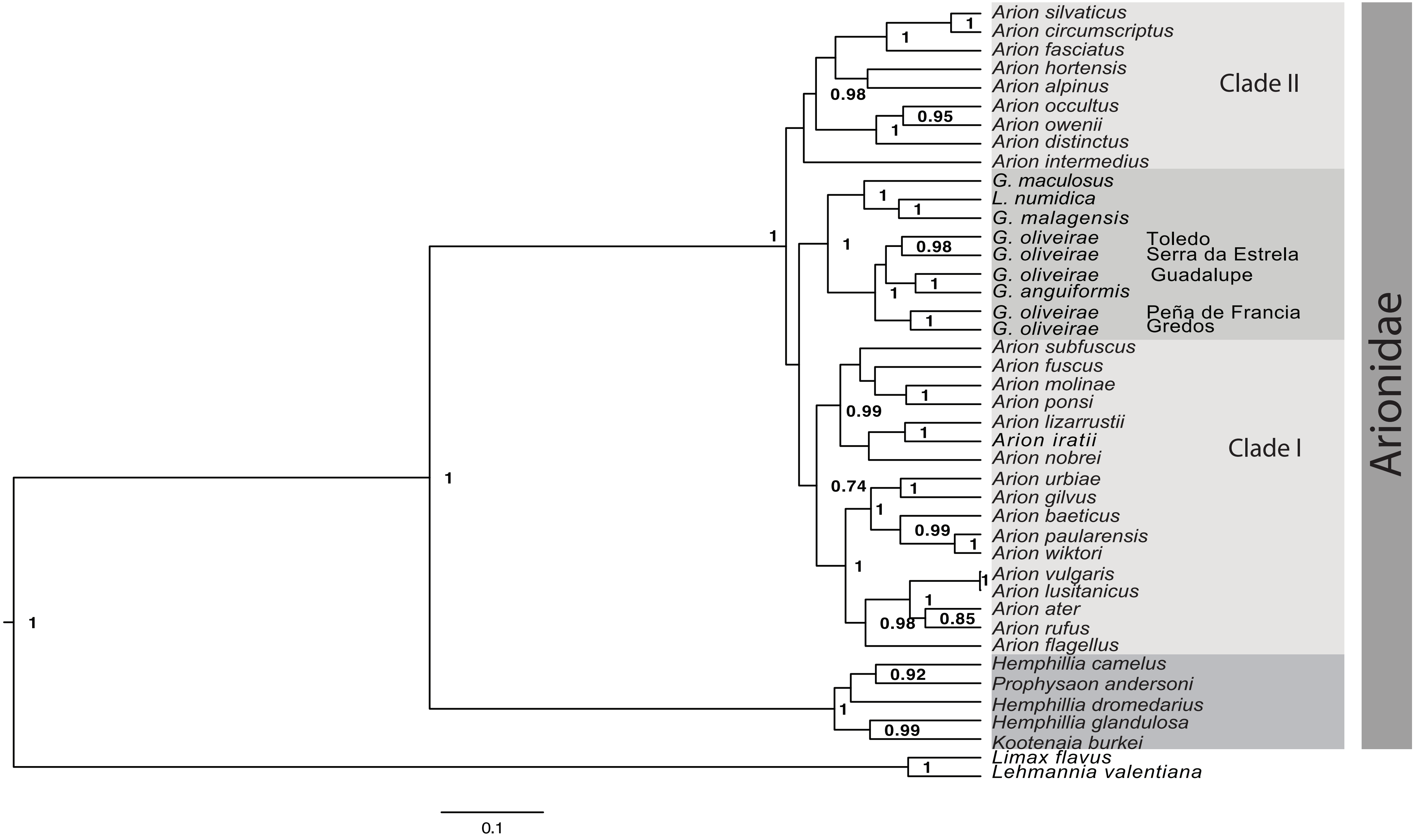

Supplementary Figure S3. BAMM phylorate plot showing diversification rate across *Arion* and *Geomalacus* species, with rates decreasing from red to blue.

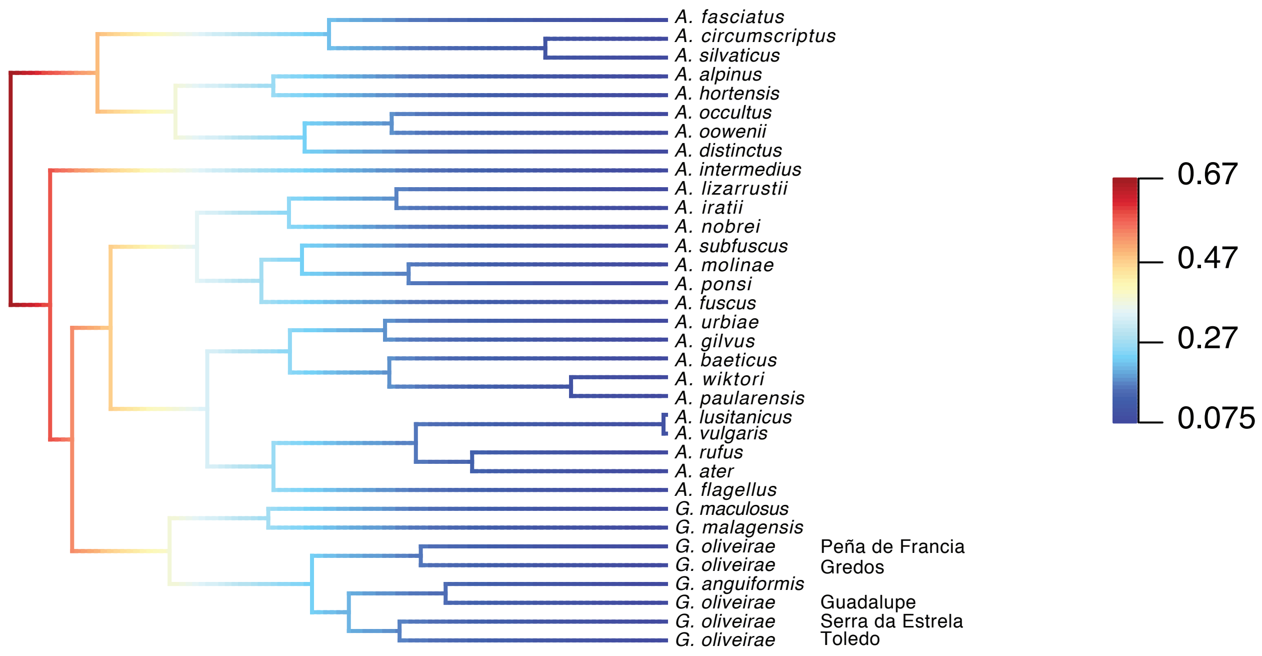

Supplement: Supplementary file 1 — Supplementary_info [file 41598_2017_16417_MOESM1_ESM.pdf]
